# A review of demodulation techniques for amplitude-modulation atomic force microscopy

**DOI:** 10.3762/bjnano.8.142

**Published:** 2017-07-10

**Authors:** Michael G Ruppert, David M Harcombe, Michael R P Ragazzon, S O Reza Moheimani, Andrew J Fleming

**Affiliations:** 1 School of Electrical Engineering and Computing, The University of Newcastle, Callaghan, NSW, 2308, Australia; 2 Department of Engineering Cybernetics, NTNU, Norwegian University of Science and Technology, Trondheim, Norway; 3 Department of Mechanical Engineering, The University of Texas at Dallas, Richardson, TX, USA

**Keywords:** amplitude estimation, atomic force microscopy, amplitude modulation, digital signal processing, field-programmable gate array

## Abstract

In this review paper, traditional and novel demodulation methods applicable to amplitude-modulation atomic force microscopy are implemented on a widely used digital processing system. As a crucial bandwidth-limiting component in the *z*-axis feedback loop of an atomic force microscope, the purpose of the demodulator is to obtain estimates of amplitude and phase of the cantilever deflection signal in the presence of sensor noise or additional distinct frequency components. Specifically for modern multifrequency techniques, where higher harmonic and/or higher eigenmode contributions are present in the oscillation signal, the fidelity of the estimates obtained from some demodulation techniques is not guaranteed. To enable a rigorous comparison, the performance metrics tracking bandwidth, implementation complexity and sensitivity to other frequency components are experimentally evaluated for each method. Finally, the significance of an adequate demodulator bandwidth is highlighted during high-speed tapping-mode atomic force microscopy experiments in constant-height mode.

## Introduction

Amplitude modulation is one of the oldest forms of modulation in analog communication systems, mostly due to its simplicity of implementation [1]. Not only is the modulation principle used in many forms of scientific instruments and sensors [2–4], but numerous fields of research also rely on the improved detection sensitivity made available by this technique [5–7].

While the invention of the atomic force microscope (AFM) [8] in the late 1980s had little to do with modulation to begin with, a fundamental prerequisite was given by the nonlinear tip–sample interaction force. With the advent of dynamic imaging modes [9], in which the microcantilever is excited at one of its resonance frequencies, the foundation for transmitting information via modulation was established. These imaging modes are especially suitable for the investigation of delicate matter and biological samples because of the low tip–sample forces [10] and have led to the instrument establishing itself as a key enabling technology for the nanoscale analysis of objects and materials properties for both research and industry [11–12].

Dynamic operating modes of the AFM can map the surface topography of a specimen with high spatial resolution by scanning a sharp tip located at the end of an actively driven microcantilever over the surface of a sample. Due to the nonlinear tip–sample forces acting on the cantilever, a feedback loop has to be employed in order to maintain a fixed setpoint with respect to the sample; the controller performs disturbance rejection by commanding a nanopositioner in its vertical direction. As the high-frequency cantilever deflection signal cannot be controlled directly, low-frequency measurables such as the change in oscillation amplitude in amplitude-modulation AFM [11] have to be employed. Other feedback variables such as the shift in cantilever resonance frequency in frequency-modulation AFM [13] or the phase shift in phase-modulation AFM [14] have also been used. Situated at the heart of these dynamic methods, a demodulator is employed to estimate amplitude and phase of the cantilever deflection signal.

A number of demodulation techniques can be found in the existing literature, some of which have found regular use in commercial AFM systems. The performance metrics, tracking bandwidth and sensitivity to other frequency components, are especially important in high-speed [15–18] and multifrequency AFM [19] applications. As the tracking bandwidth directly affects the achievable scan rate, it should be maximized. However, this also increases the noise bandwidth. On the other hand, in multifrequency AFM applications, the sensitivity to other frequency components is of greatest concern. These applications may include multiple eigenmode contributions [20–22], higher harmonics [23–25], and multi-tone near-resonance frequency components [26–28].

For instance, RMS-to-DC conversion [29] is low in implementation complexity and can achieve high tracking bandwidth, but it is sensitive to other frequency components. In contrast, the lock-in amplifier [30–32] is a narrow-band technique that has been adopted as the industry-wide standard in commercial AFMs, since it is insensitive to other frequency components but is limited in tracking bandwidth.

Inspired by image-rejection mixers [33] and modulated–demodulated control [34], a high-bandwidth lock-in amplifier was recently proposed and implemented to improve upon this constraint [35]. However, the method is still ultimately limited by the low-pass filters that are required to account for residual phase mismatches.

For high-speed AFM applications, as required for the study of fast biological processes [36–37], the above methods are not suitable and have led to the development of fast single-wave detectors in the form of the peak-hold method [38–39] and coherent demodulator [40–43]. The latter is an all-digital lock-in amplifier where the characteristic low-pass filter is replaced by a precise numerical integration scheme. While these methods can yield fast estimates with low latency, they may not be suitable for multifrequency AFM methods where non-integer multiples of the fundamental frequency are present in the deflection signal.

The demand for a high tracking bandwidth while maintaining insensitivity to additional frequencies in the signal has motivated the development of filters such as the time-varying Kalman filter [44] and Lyapunov filter [45–46]. These methods are based on a linear parametric model of the cantilever deflection signal and were shown to be extendable for the estimation of multiple frequencies for multifrequency AFM [47–49].

Observer-based approaches have also been investigated to provide an alternative feedback signal other than the estimated amplitude. For instance, if an observer is constructed from the free-air model of the cantilever, the innovation signal (error signal between measurement and model output) will contain information of the disturbance profile during the transient response of the cantilever [50–53]. In addition, it was shown that the tip–sample force can be estimated directly by assuming it takes the form of an impulse train [54]. In this way, the tip–sample force is estimated directly, thus potentially enabling high-bandwidth *z*-axis control by relying on feedback from the force estimate instead of from the cantilever oscillation amplitude.

This article aims to provide a rigorous experimental comparison of the most commonly used demodulation methods for amplitude-modulation AFM over their entire tracking bandwidth range. The methods considered are the lock-in amplifier, high-bandwidth lock-in amplifier, Lyapunov filter, Kalman filter, RMS-to-DC conversion (moving-average filter and mean absolute deviation computation), peak detector and coherent demodulator. To make a fair comparison, a widely used digital signal processing system (LabVIEW) is used and the implementations are unified to a common sample rate. The performance metrics are tracking bandwidth, implementation complexity, sensitivity to other frequency components and total integrated noise of the amplitude estimate as a function of the tracking bandwidth. The experimental analysis is concluded by high-speed constant-height tapping-mode AFM experiments which highlight the case where the demodulator is the bandwidth bottleneck in the *z*-axis feedback loop.

## Fundamentals of amplitude modulation and demodulation

### Modulation

A basic amplitude-modulated (double-sideband full carrier) signal is obtained by mixing a modulating signal *y_m_*(*t*) at a modulation index *M* and frequency ω*_m_* = 2π*f_m_* with a carrier signal *y_c_*(*t*) with (for the sake of brevity) unity amplitude, phase 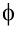, and frequency ω*_c_* = 2π*f_c_* such that

[1]
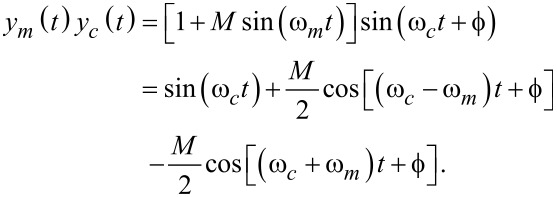


**Figure 1:**
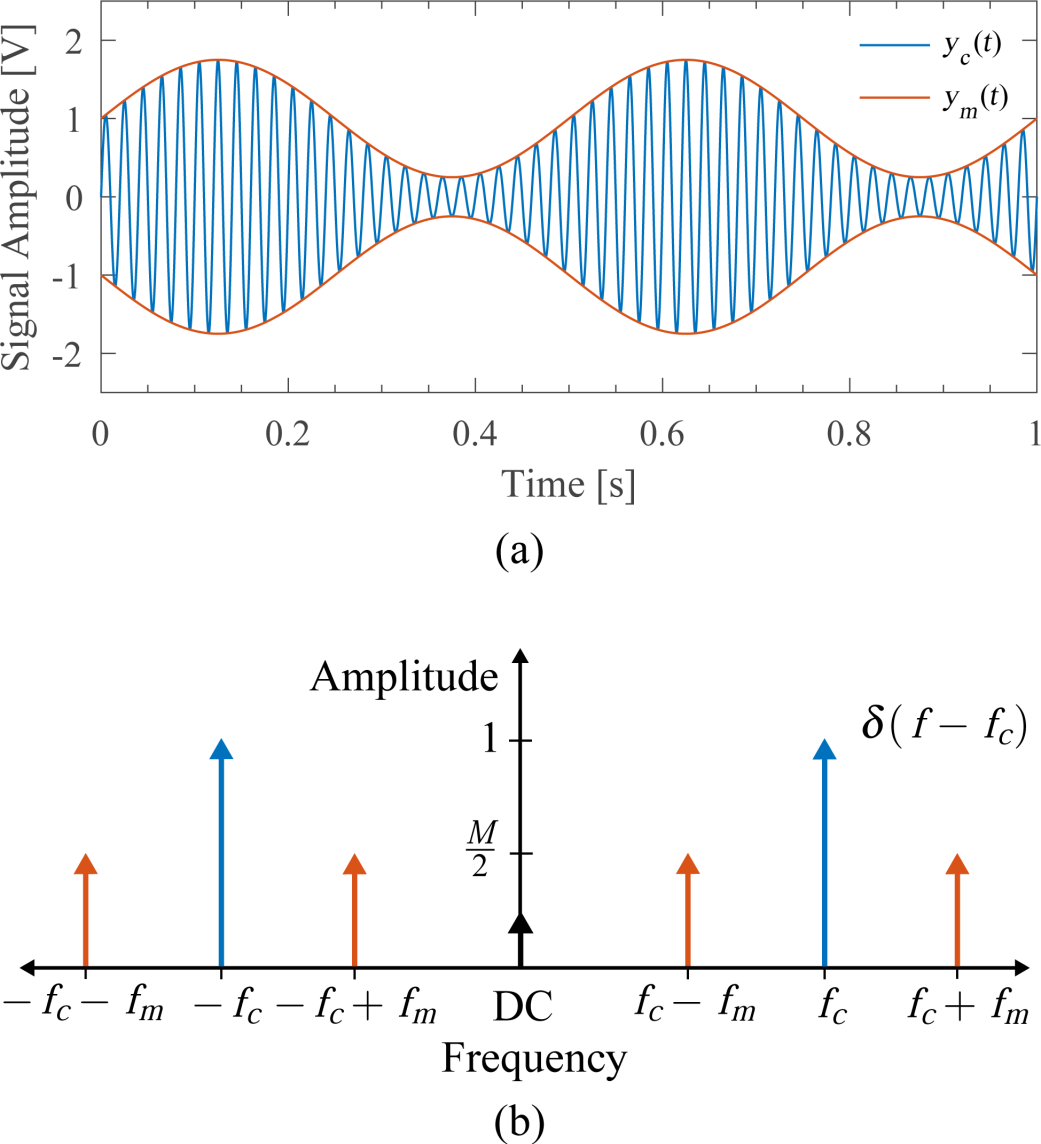
(a) Time-domain example of an amplitude modulated signal with carrier frequency *f_c_* = 50 Hz, modulating frequency *f_m_* = 5 Hz, and modulation index *M* = 0.75 and (b) its double-sided amplitude spectrum. A small residual DC offset is also shown for the sake of completeness.

In the time domain, this process is shown in Figure 1a, where the minimum and the maximum levels attained by the amplitude-modulated signal are 1 − *M* and 1 + *M*, respectively. It can be seen from Equation 1 that the modulation process creates distinct frequency components located at *f_c_* and *f_c_* ± *f_m_*. The latter components are termed the upper and lower sidebands and are centered symmetrically around the carrier frequency for *f_m_* *< f_c_*, illustrated in Figure 1b. As the modulating frequency increases, these sidebands move away from the carrier until they appear at DC and at 2*f_c_* for the limit where *f_m_* = *f_c_*. For the case where *f_m_* *> f_c_*, *y*(*t*) resembles a distorted wave with sidebands located at *f_m_* ± *f_c_* and can therefore no longer be considered an amplitude-modulated signal because the sidebands are no longer symmetrically located around the carrier frequency. For the application in AFM, this case is practically irrelevant as it corresponds to amplitude changes appearing faster than the tapping frequency.

### Demodulation

**Figure 2:**
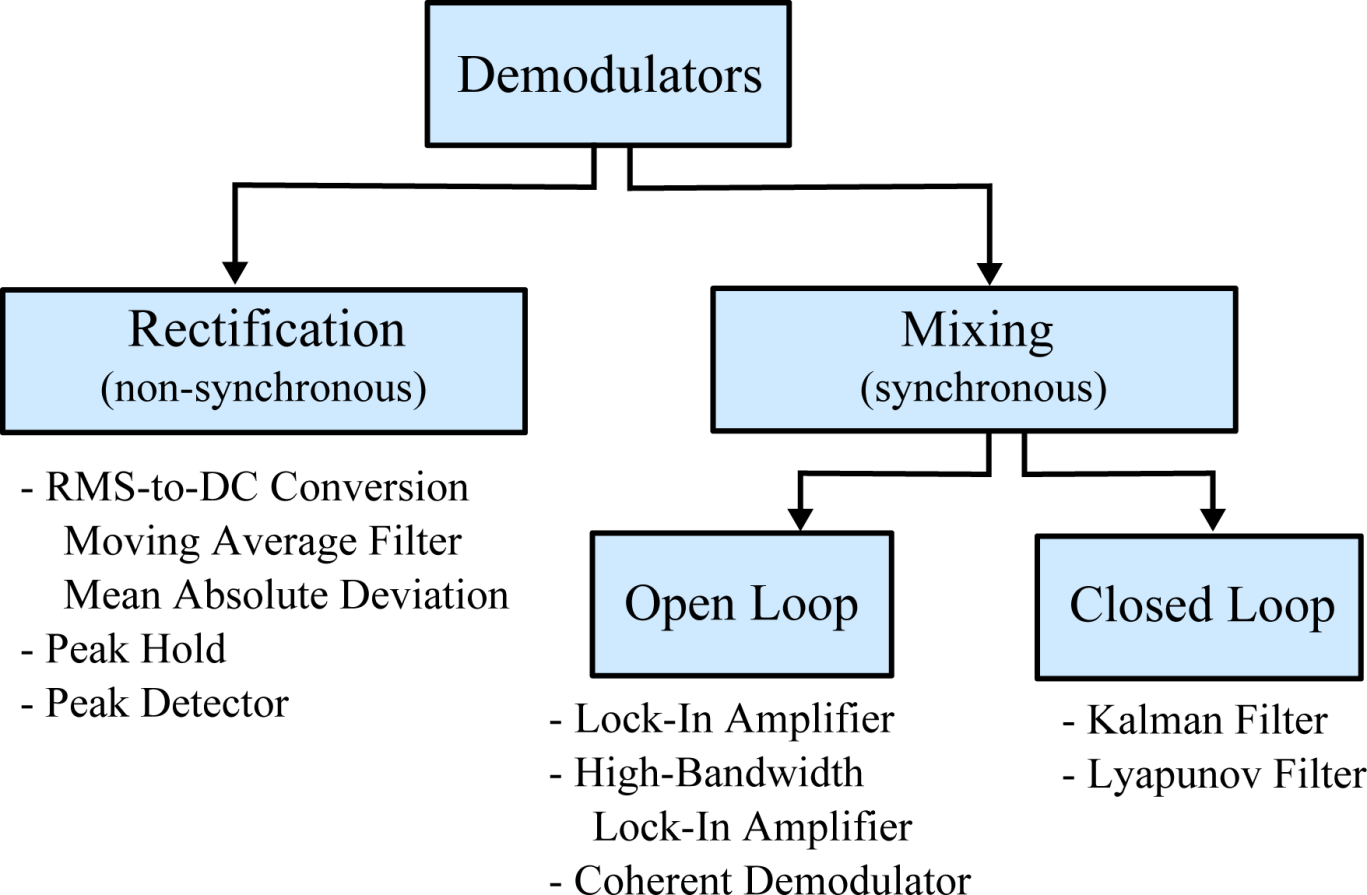
Classification of demodulation methods discussed in this paper.

The process of demodulation always requires a nonlinear operation on a signal in order to estimate a baseband signal proportional to the modulation of the carrier. Based on this nonlinearity, the demodulation methods can be broadly classified as methods using rectification (non-synchronous detection) and methods using mixing with a reference oscillator signal (synchronous detection). For demodulators of the latter class, the reference signal can be either a square wave, most commonly used for analog implementations, or a sinusoid, most commonly used for digital implementations as is the case in this paper. Within the class of demodulators using mixing, further classification can be made based on how the 2*f_c_* component from the mixing process is filtered out. While the open-loop methods rely on either general or numerically precise low-pass filters, the closed-loop methods employ feedback of the parameterized signal states to eliminate this component. An overview of the demodulator classification is shown in Figure 2. As will be discussed in the course of this paper, each class has distinct properties with regards to tracking bandwidth, implementation complexity and sensitivity to other frequency components.

The linear parameterization used by the demodulation techniques based on mixing is derived from a sine wave with known carrier frequency ω*_c_*, unknown amplitude *A* = *A*(*t*) and unknown phase 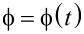 of the form

[2]
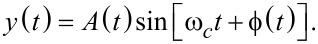


The signal can be rewritten as a sum of its quadrature and in-phase components by applying trigonometric identities in order to obtain a linear parameterization (the time dependency for slowly changing parameters are left out for the sake of readability)

[3]
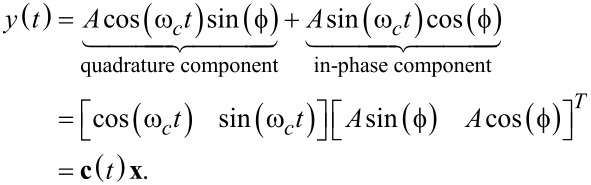


As we will make frequent reference to this parameterization, the entries of the vector **c**(*t*) are termed the quadrature and in-phase sinusoids and the entries of the state vector


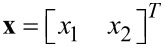


are termed the quadrature and in-phase states. In this form, amplitude and phase can be directly calculated as

[4]
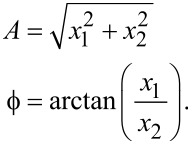


### Performance metrics

The performance metrics used for the comparison of the demodulation methods are implementation complexity, tracking bandwidth, sensitivity to other frequency components and total integrated noise of the amplitude estimate as a function of the tracking bandwidth. The implementation complexity is qualitatively evaluated based on the maximum free-running sample rate achieved by the digital signal processing system. Where applicable, latencies arising from fixed time-delays in the implementation of the methods are highlighted. The tracking bandwidth is defined as the frequency *f*_−3dB_, at which the amplitude estimate drops by −3 dB. This figure of merit is important to determine both the speed of convergence and the amount of noise suppression in the estimate. This relationship is clearly identified by plotting the total integrated noise of the amplitude estimate against the tracking bandwidth for a known input noise density. Lastly, the sensitivity to other frequency components is evaluated to determine the ability of each method to filter out any signal at frequencies other than the carrier frequency of interest.

## Review of demodulation methods

### Lock-in amplifier

The lock-in amplifier [30–32] mixes the input signal (Equation 2) with in-phase and quadrature sinusoids to obtain

[5]
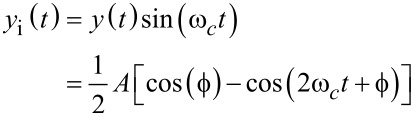


and

[6]
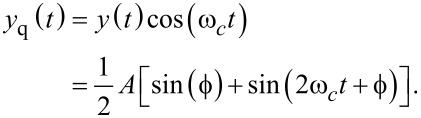


**Figure 3:**
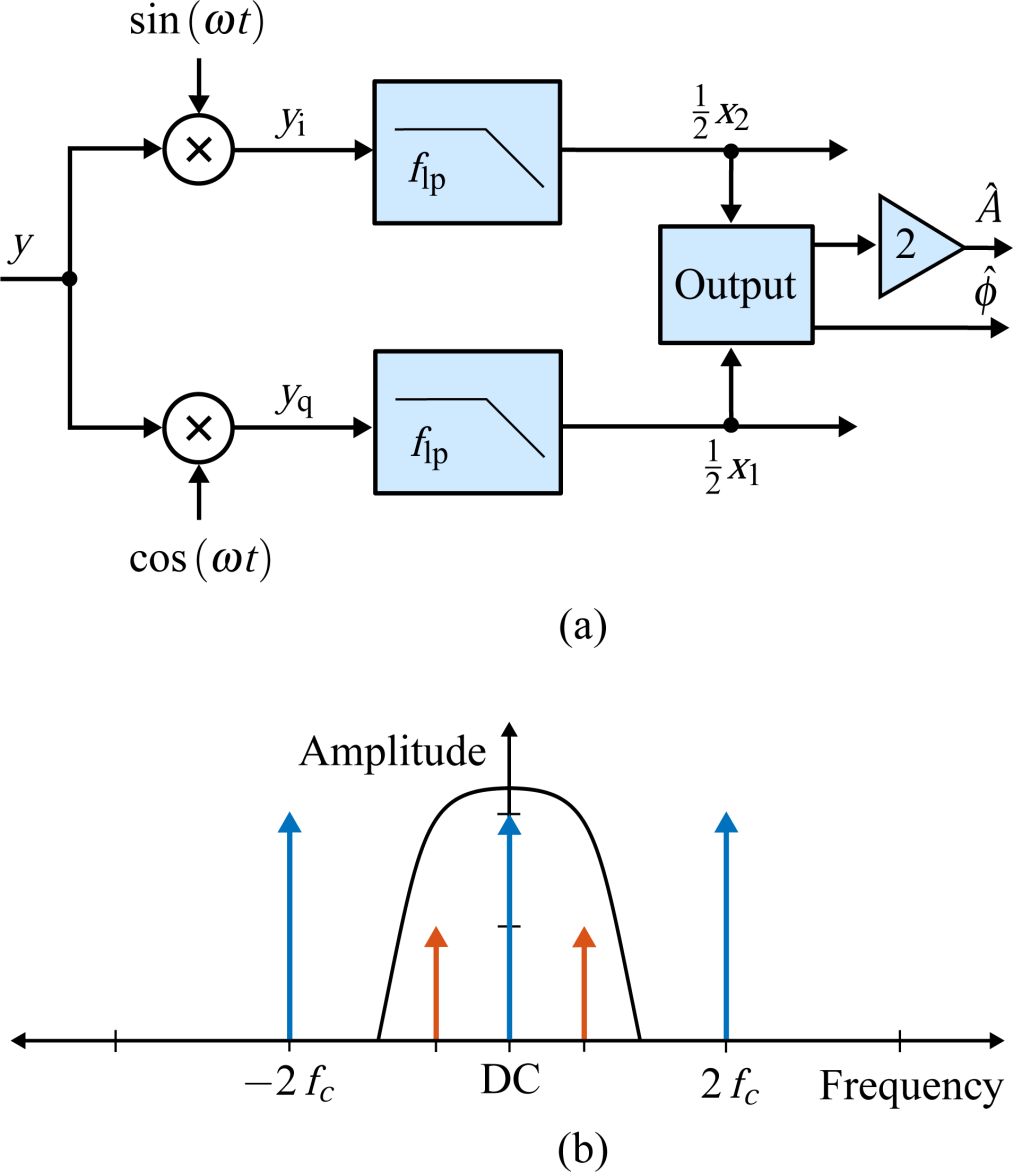
(a) Functional block diagram of the lock-in amplifier implementation and (b) illustrative double-sided amplitude spectrum of the signal after mixing.

From Equation 5 and Equation 6, it can be seen that the mixing process generates harmonics at 2*f_c_*, which need to be removed by employing a low-pass filter with 
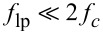
, as illustratively shown in Figure 3b. Further, any residual DC offset in the input signal will generate a harmonic at *f_c_*, which is the reason why lock-in amplifiers should always be AC-coupled. The order and cut-off frequency of the low-pass filter directly determines the tracking bandwidth and hence the noise performance. For instance, in order to limit the ripple to 1% of the signal, a −40 dB suppression of the 2*f_c_* component is required. A 2nd-order low-pass filter would achieve this by limiting the bandwidth to approximately a decade below the carrier frequency.

A lock-in amplifier described by Equations 4–6 can be realized digitally with a direct digital synthesizer (DDS) to create the reference sine and cosine signals, two multipliers, two low-pass filters, and an output block with square-root functionality and an arctan calculation method such as polynomial approximation or the CORDIC algorithm [55] to calculate the phase [43]. Such an implementation is schematically shown in the block diagram in Figure 3a, where the output block represents Equation 4. A factor of two is necessary to obtain the correct amplitude scaling.

### High-bandwidth lock-in amplifier

The high bandwidth lock-in amplifier is a novel extension to the standard lock-in amplifier technique, which employs phase cancellation to precisely cancel the 2*f_c_* term [35]. The method is inspired by radio frequency image rejection mixers [33] and modulated–demodulated control [34,56]. Compared to the standard lock-in amplifier, this demodulation scheme essentially requires two additional multipliers, which operate on the input signal shifted by 90°

[7]
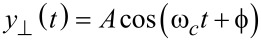


to form the respective output products

[8]
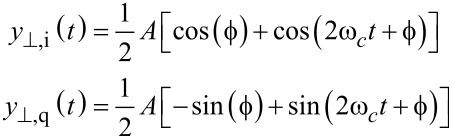


which are added to the output products of the LIA mixers to exactly cancel the 2*f_c_* components

[9]
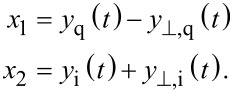


**Figure 4:**
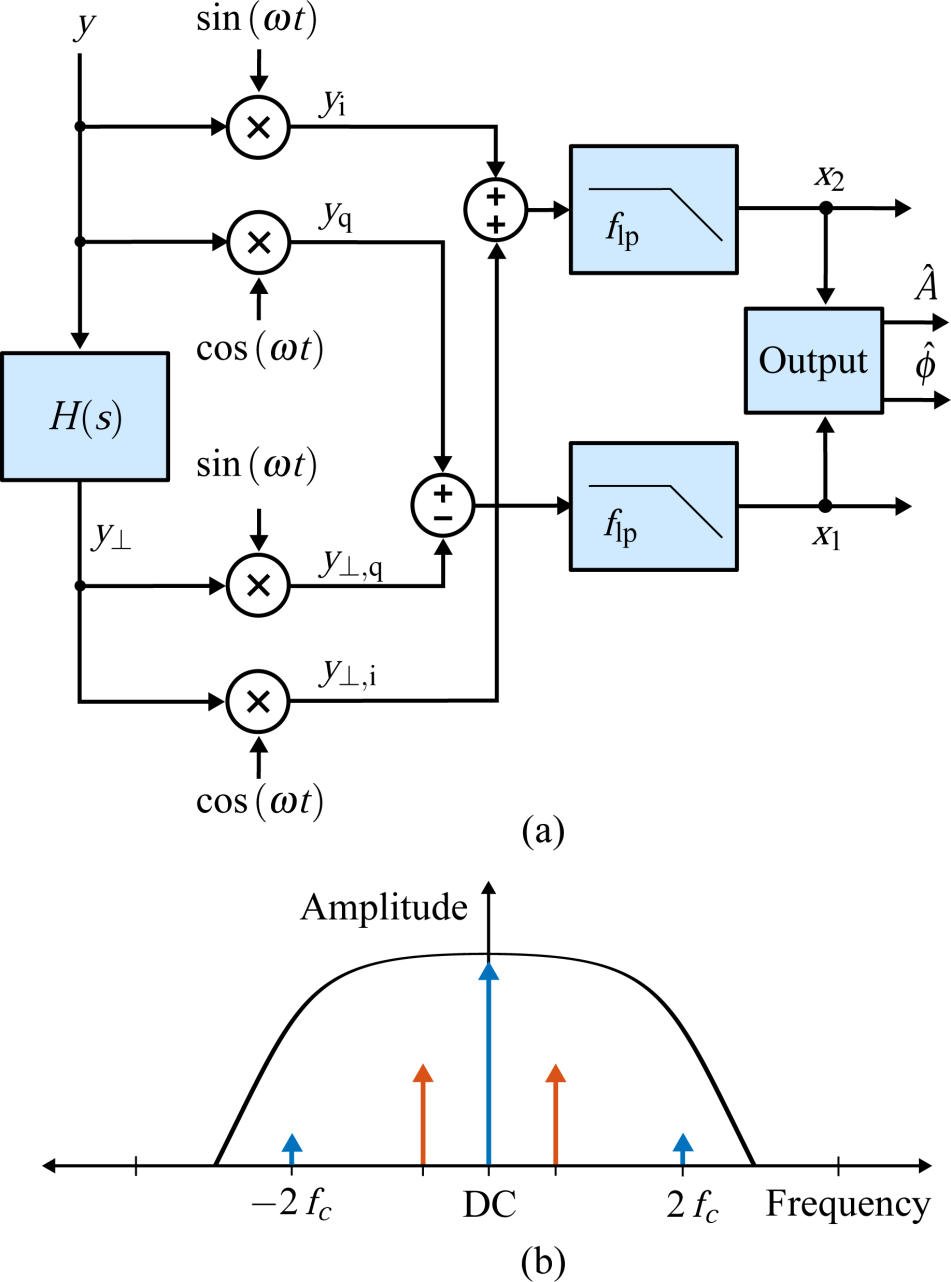
(a) Functional block diagram of the high-bandwidth lock-in amplifier implementation and (b) illustrative double-sided amplitude spectrum of the signal after the summing stage.

Although an analog implementation is possible [35], in practice imperfect phase cancellation due to circuit mismatches still requires post-mixing low-pass filters. However, as the 2*f_c_* terms are heavily attenuated, the bandwidth of the filters can be increased. This is illustrated in Figure 4b.

In a digital implementation, the standard lock-in amplifier implementation has to be extended with two additional multipliers, two summing stages and a 90° phase-shift block *H*(*s*) as shown in Figure 4a. Such an operation can be realized with a Hilbert transform filter or an all-pass filter tuned to the carrier frequency [57]. Amplitude and phase are recovered by employing the output Equation 4 without an additional scaling factor.

### Kalman filter

**Figure 5:**
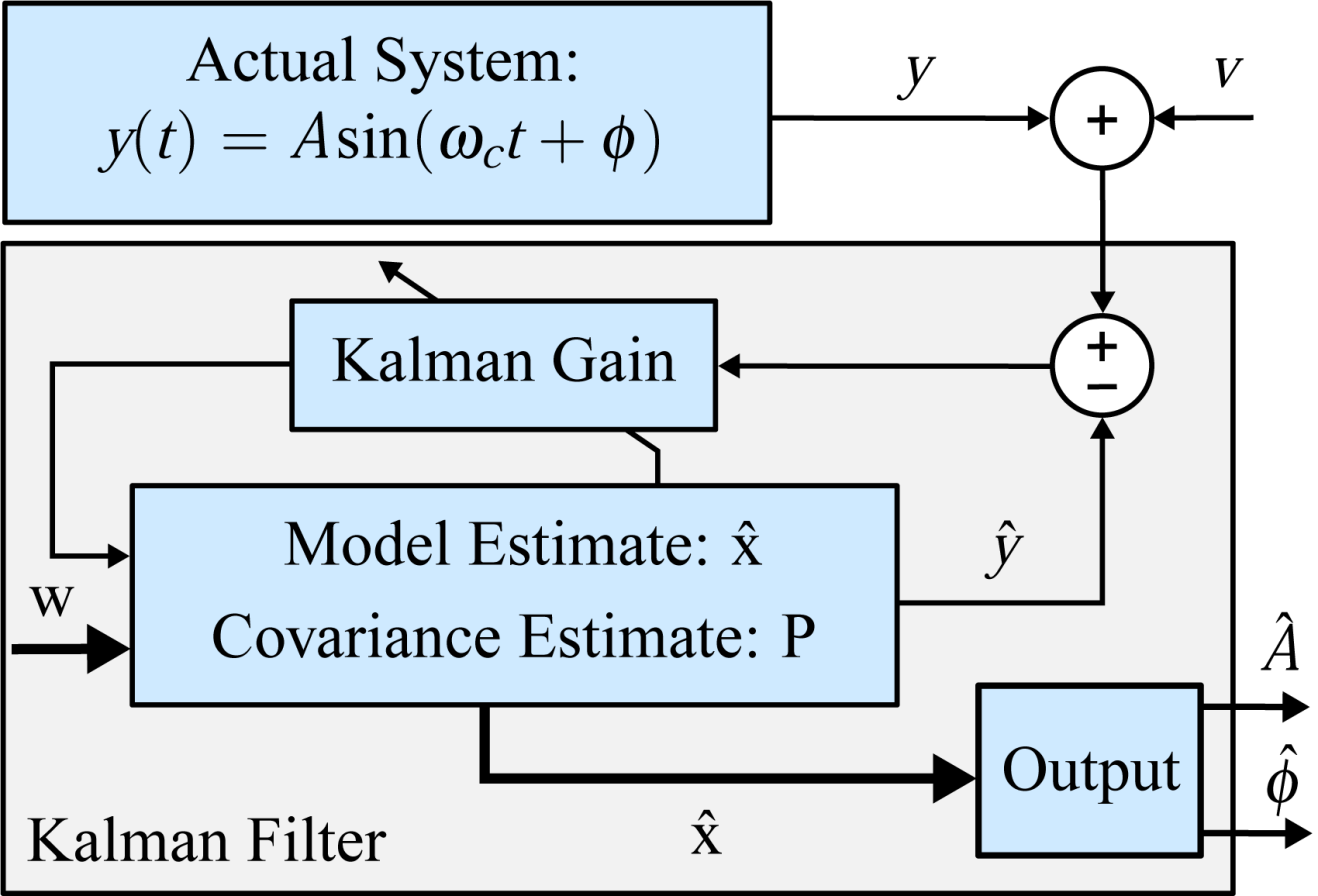
General block diagram of the Kalman filter for demodulation. Thick lines indicate vector-valued signal paths and thin lines indicate scalar signal paths.

The Kalman filter [58] can generally be considered as a recursive algorithm that makes the best possible trade-off between modeled and measured information to estimate unknown variables of a process. Specifically, if the modeling error and the noise in the measurement can be considered to have a Gaussian distribution, the Kalman filter is the minimum variance estimator in the least-squares sense [59]. Typical uses of the Kalman filter include sensor fusion, smoothing noisy data and estimation of internal states in numerous applications ranging from navigation, economics and signal processing [60]. Characteristic and fundamental to its working principle is the existence of a linear system model that describes the dynamics to be estimated and the presence of feedback generated from the Kalman gain, which dictates the rate of convergence. This structure is schematically shown in Figure 5.

A linear time-invariant (LTI) state-space model of the signal described by Equation 2 can be obtained by choosing 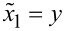 and 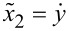 as the state variables and 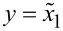 as the output to yield

[10]
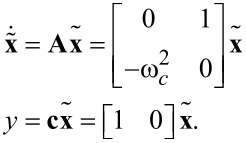


However, due to the sparse nature of the resulting dynamic matrix **A**, especially when modeling higher resonance frequencies, this model is generally ill-conditioned for the use in an observer such as the Kalman filter, which requires an inversion. This problem is circumvented by applying a time-variant transformation 
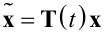
 with [44]

[11]
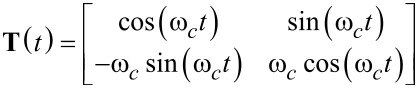


such that a time-varying but numerically well-conditioned state-space representation is obtained. This constitutes the process model of the Kalman filter, which in its discretized form is given as

[12]
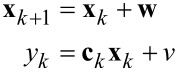


[13]
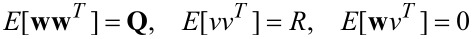


where 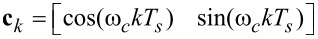, *T_s_* is the sample period, and **w** and *v* are the process noise and the measurement noise with their respective covariance matrices **Q** and *R*. A similar system description was successfully used in tracking power system voltage phasors [61]. In this representation, the states *x*_1_*_,k_* and *x*_2_*_,k_* are assumed to be random variables describing the quadrature and in-phase states of Equation 3. Moreover, **Q** determines the amount of uncertainty in the model (Equation 12) and *R* the amount of noise in the measurement. If *R* is chosen to be the standard deviation σ of the Gaussian noise in the sensor signal *y*(*t*) (*R* = σ^2^), then **Q** remains the only tuning variable to set the tracking bandwidth of the estimated amplitude and phase. The recursive implementation follows the standard equations of the Kalman filter [62–63] and are stated in Appendix A. Amplitude and phase are recovered by employing the output equations in Equation 4.

### Lyapunov filter

The Lyapunov filter is conceptually related to the Kalman filter in the sense that it uses feedback to correct the estimated quadrature and in-phase states of Equation 3 of the linear parameterization of the signal (Equation 2). Compared to the Kalman filter, it is significantly less computational expensive as it does not require the computation of a covariance matrix to determine the feedback gain. Instead, the gain is a predetermined constant parameter that is related to the Kalman gain for certain conditions [46].

In the literature about adaptive control, the Lyapunov filter is regarded as an online adaptive estimator for which the estimation error relates to the parameter error through a strictly positive real (SPR) transfer function *W*(*s*) [64]. This SPR property is exploited in designing the update law via a Lyapunov stability proof to show boundedness of the error, hence the name. An additional persistency of excitation property [64] guarantees exponential convergence of the parameters. The estimator can be written in the compact form [45–46]

**Figure 6:**
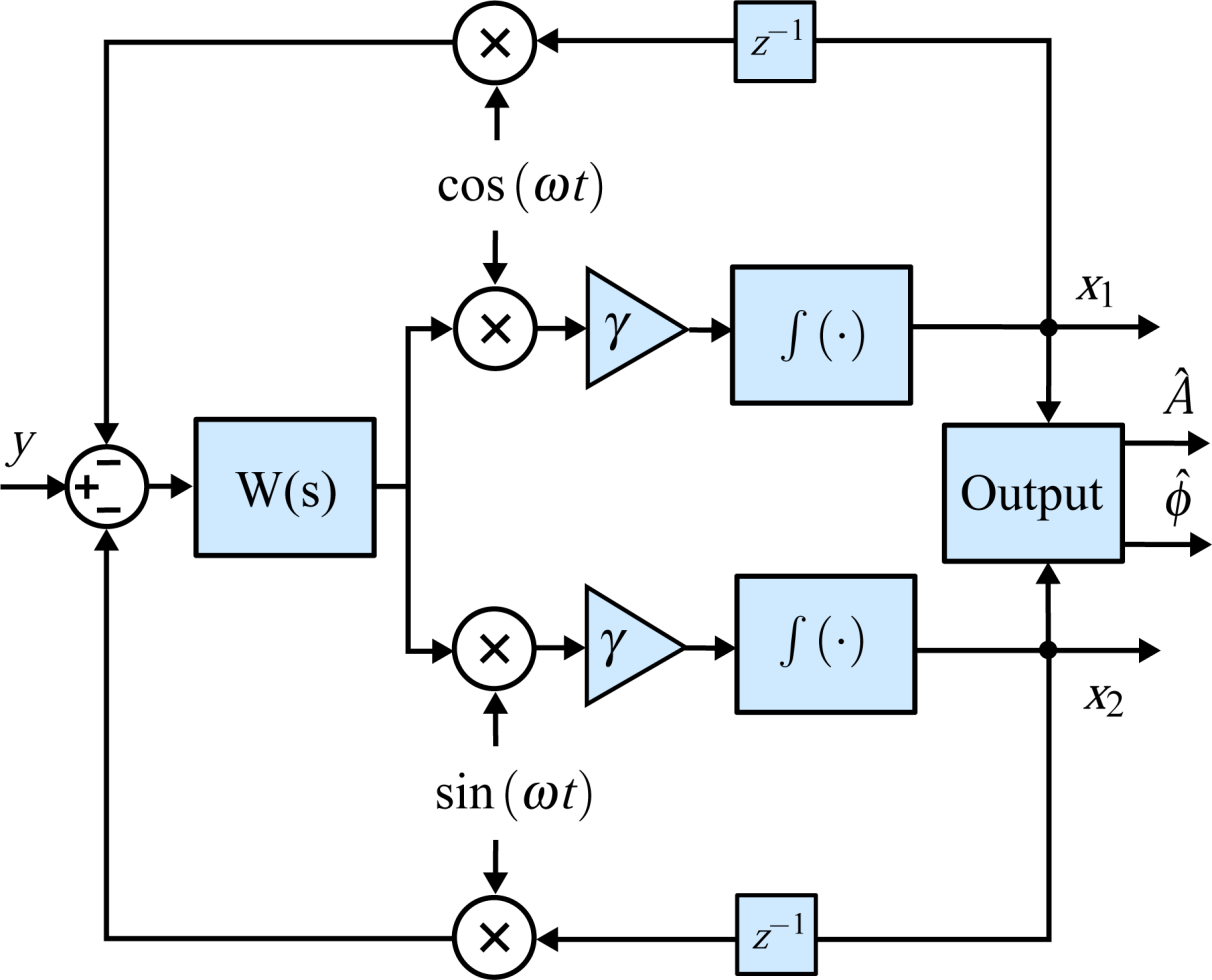
Functional block diagram of the Lyapunov filter implementation.

[14]
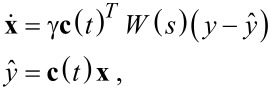


where γ is the constant gain parameter for tuning the bandwidth and 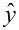 is the estimated signal. For simplicity, *W*(*s*) can be assumed to be a constant 1 as any other assumption will limit the tracking bandwidth [46]. A digital implementation requires a DDS, four multipliers, two gain blocks, a discrete filter and two discrete integrators but can also be realized with scalar operations as shown in Figure 6. The amplitude and phase are recovered by employing the output equations in Equation 4.

### RMS-to-DC conversion methods

One of the easiest forms of amplitude estimation is RMS-to-DC conversion. The root-mean-square (RMS) value *y*_rms_ of a sinusoidal signal *y*(*t*) with period *T* is proportional to the amplitude of the signal and is defined as

[15]
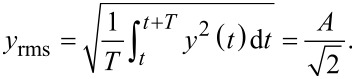


True RMS-to-DC conversion can be achieved in analog using either direct or indirect computation. The direct method performs the squaring, averaging and square-root functionality using multipliers and operational amplifiers. While the benefit of direct computation is a large bandwidth, it has a limited dynamic range due to the squaring stage [29]. Indirect computation alleviates this problem by employing feedback and division of the average output, which significantly improves the dynamic range but comes at the expense of tracking bandwidth. A number of direct and indirect analog true RMS-to-DC converters are available commercially. For a purely sinusoidal signal, the output of these methods are proportional to the oscillation amplitude. However, biased amplitude estimates are obtained when additional frequency components are present in the signal.

#### Moving average filter

**Figure 7:**
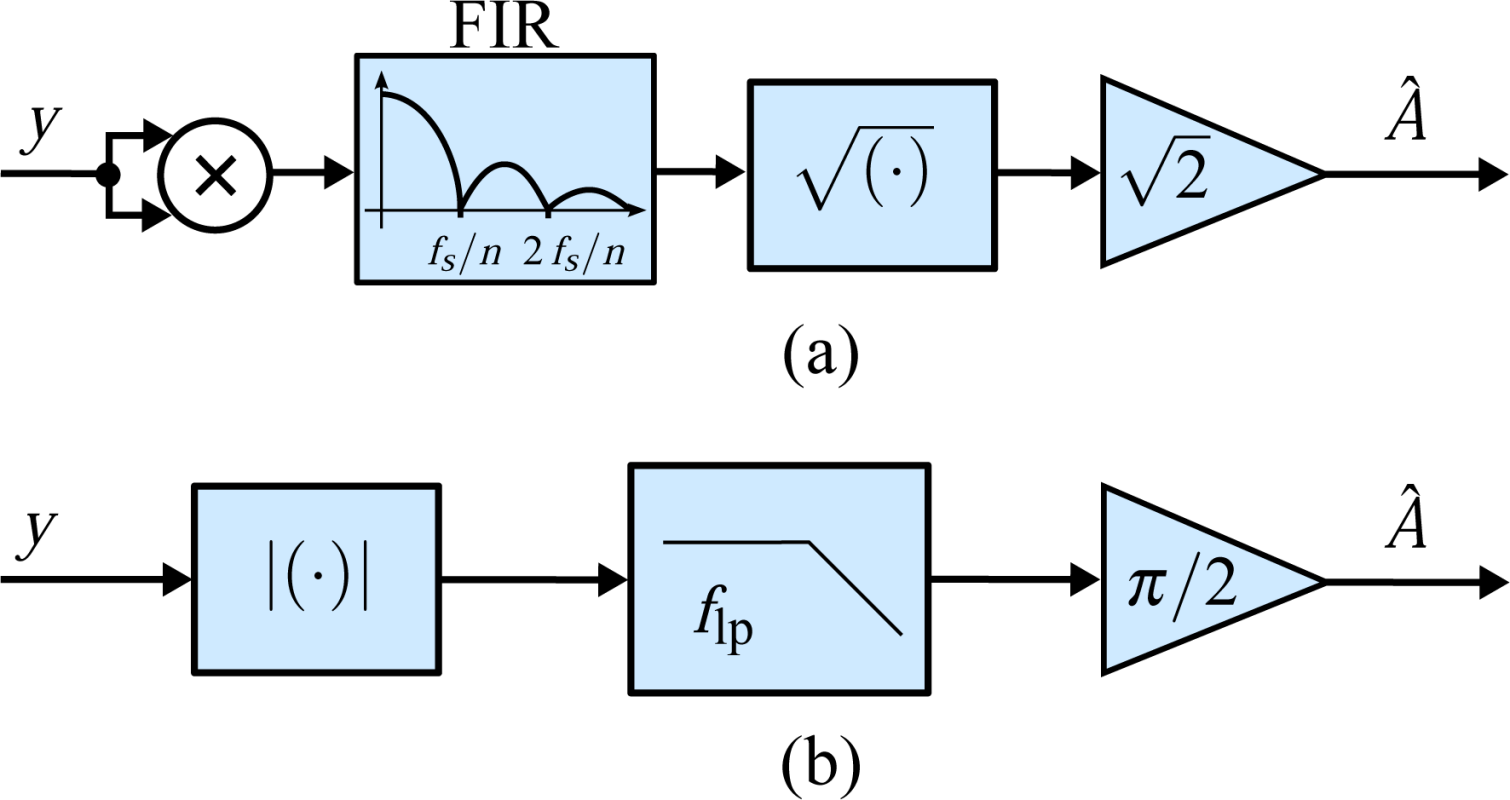
Functional block diagram of (a) moving average filter and (b) mean absolute deviation measurement to perform digital RMS-to-DC conversion.

Equation 15 can be implemented digitally by obtaining the moving average using a finite impulse response (FIR) filter [57] of the squared input signal and passing it through a subsequent square-root stage. The integration period *T* in Equation 15 is related to the length of the (*n* + 1)-tap moving average FIR filter as *T* = *n*/*f_s_*, which dictates the tracking bandwidth of this approach. The number of samples *n* should be a half-period integer multiple of the sample rate *f_s_*. A functional block diagram of this implementation is shown in Figure 7a. As a true RMS-to-DC converter, the output needs to be scaled by 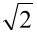 to obtain the amplitude as evident from Equation 15. This method has increasing latency for decreasing tracking bandwidth.

#### Mean absolute deviation

In tapping-mode AFM, RMS-to-DC conversion was typically performed using a precision rectifier circuit and a low-pass filter [36]. Strictly, this is not RMS-to-DC conversion but mean absolute deviation [29], which calculates the AC average of the waveform 2*A*/π. In a digital implementation, this circuit can be realized with an absolute value block representing the rectifier, a low-pass filter and an output scaling factor of π/2. The functional block diagram of this implementation is shown in Figure 7b.

### Peak hold and peak detector method

**Figure 8:**
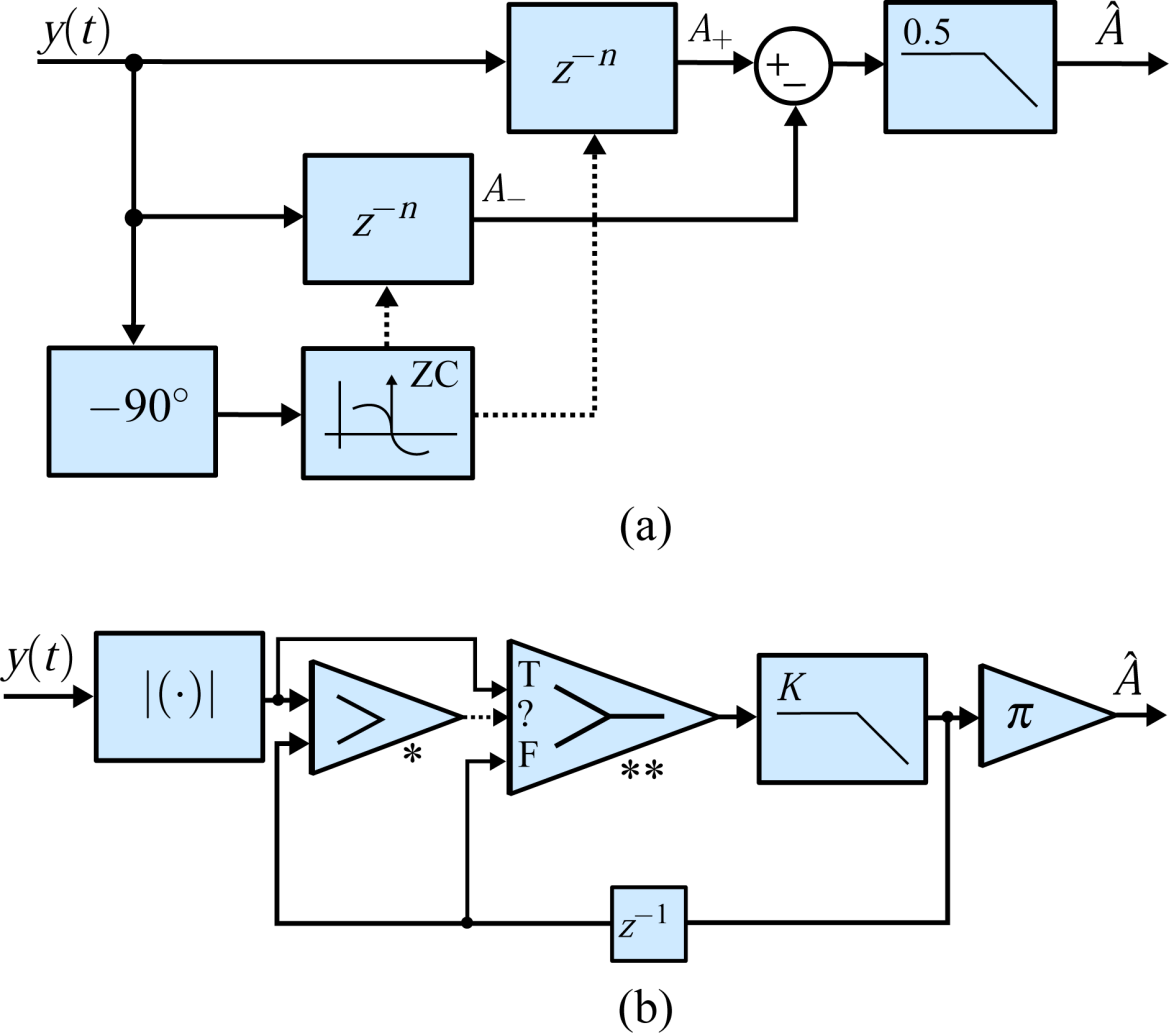
Functional block diagram of (a) the peak hold method and (b) the modified peak hold method based on a peak detector implementation alleviating the sample frequency limitation. The blocks labeled * and ** follow the LabVIEW-specific layout and represent the “greater” and triggered selector functionality.

The peak hold technique [36,38] was specifically developed for high-speed tapping-mode AFM, enabling video-rate imaging of Myosin V [15]. The analog implementation of this method comprises two sample and hold circuits to hold both the positive and negative peaks of the carrier signal for the duration of a cycle triggered by using a zero-cross comparator on the phase-shifted signal. By calculating the arithmetic mean of the outputs of the two sample and hold circuits and passing it through a low-pass filter to set the bandwidth, the output represents the amplitude of the input signal. The functional block diagram of this implementation is shown in Figure 8a.

While this technique offers high tracking bandwidth, it is more susceptible to measurement noise and other frequency components in the signal as there are only two measurements per cycle. Furthermore, the presence of harmonics will result in biased amplitude estimates and complicates the synchronization of the sample and hold circuitry. In a digital implementation, the triggering of the sample and hold blocks can be simplified with a single zero-cross comparator and knowledge of the sample frequency and carrier frequency. However, this approach requires a sufficiently high sample rate to carrier frequency ratio *m* = *f_s_*/*f_c_* such that the zero-crossing can be detected accurately. Then, knowing that the negative peak will appear at *m*/4 samples after the zero-crossing and the positive peak will appear at 3*m*/4 samples after the zero-crossing, *m* must be at least 4, or any integer multiple.

For the digital system used in this work and the chosen carrier frequency, detecting the zero-crossing with only 6 samples per cycle is infeasible. As such, a modified peak detection method is implemented that does not rely on accurate timing [45]. The block diagram is shown in Figure 8b. The method quickly tracks rising amplitudes due to the comparator and then slowly decreases the estimate based on the low-pass filter gain 0 *< K <* 1. We chose *K* = 0.5 throughout this paper and the low-pass filter is used to set the tracking bandwidth.

### Coherent demodulator

A digital low-latency, coherent demodulation method has been proposed based on mixing and post-integration over a fixed time window [40–43]. Conceptually, it is an all-digital lock-in amplifier implementation that mixes the signal to be demodulated with in-phase and quadrature sinusoids

[16]
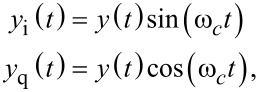


and implements the low-pass filtering of the harmonic content with a precise fixed-length numerical integration [41]. If the input signal is a pure sinusoid and the integration period *T* is chosen to be an integer multiple of the drive signal period, *T* = *mT_c_*, the integrals over *y*_i_(*t*) and *y*_q_(*t*) evaluate exactly to the in-phase and quadrature states

[17]
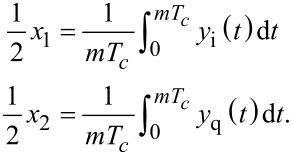


**Figure 9:**
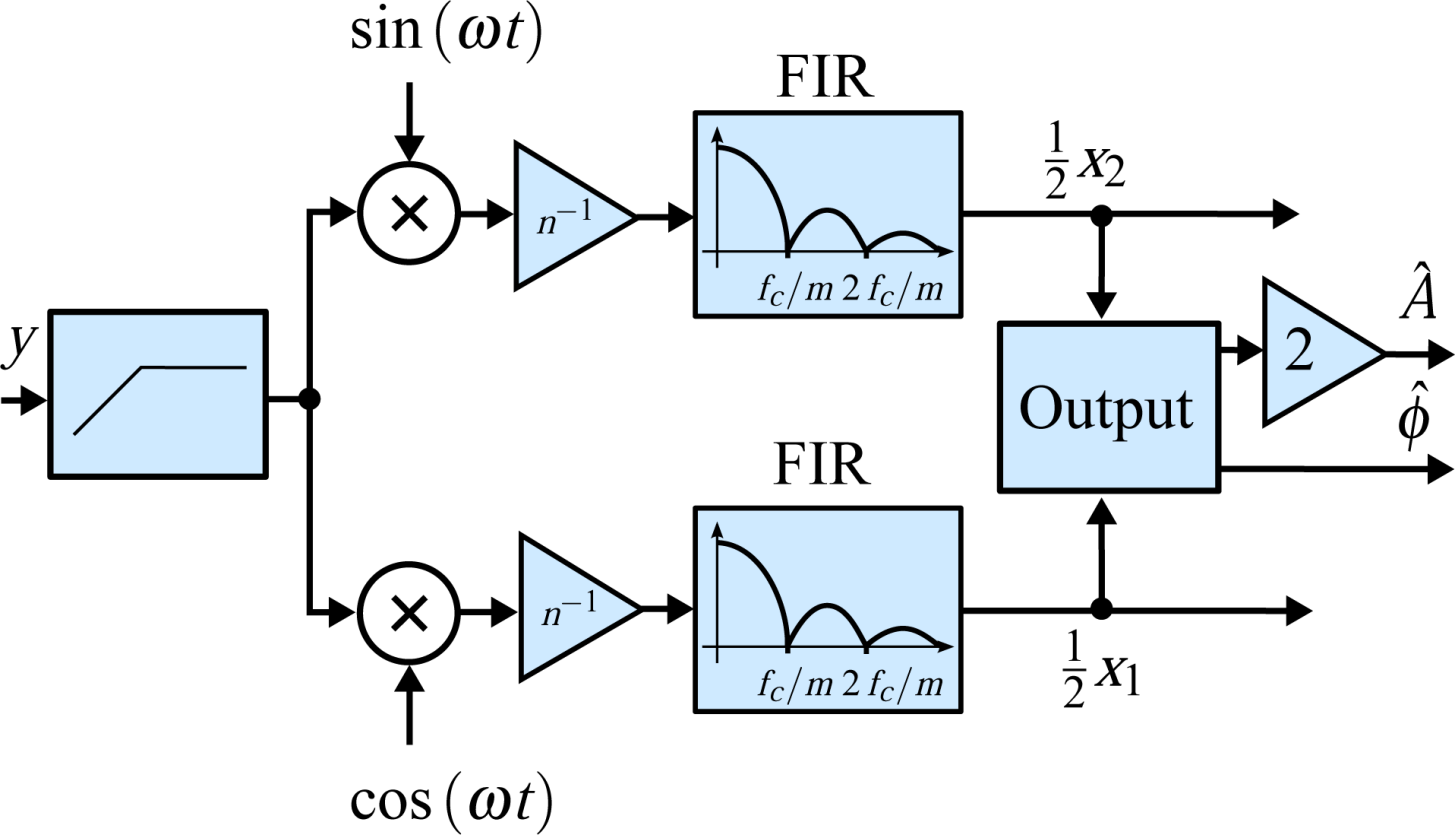
Functional block diagram of the coherent demodulator implementation.

The functional block diagram of this implementation is shown in Figure 9. Of particular importance is the timing and integration length of this approach. For Equation 17 to hold, the integration period must be an integer multiple of the sampling period *nT_s_* = *mT_c_*, where *n* is the number of samples in the integration. However, for an arbitrary carrier frequency the ratio *f_s_*/*f_c_* is rarely an integer making this condition hard to meet. Therefore, a practical solution is to find the smallest *n* such that *nT_s_* ≤ *mT_c_* ≤ (*n* + 1)*T_s_* and performing a partial integration over the last sampling interval [41]. Such precise control over the integration period is achievable in digital systems, however, the implementation of this method is still challenging.

**Figure 10:**
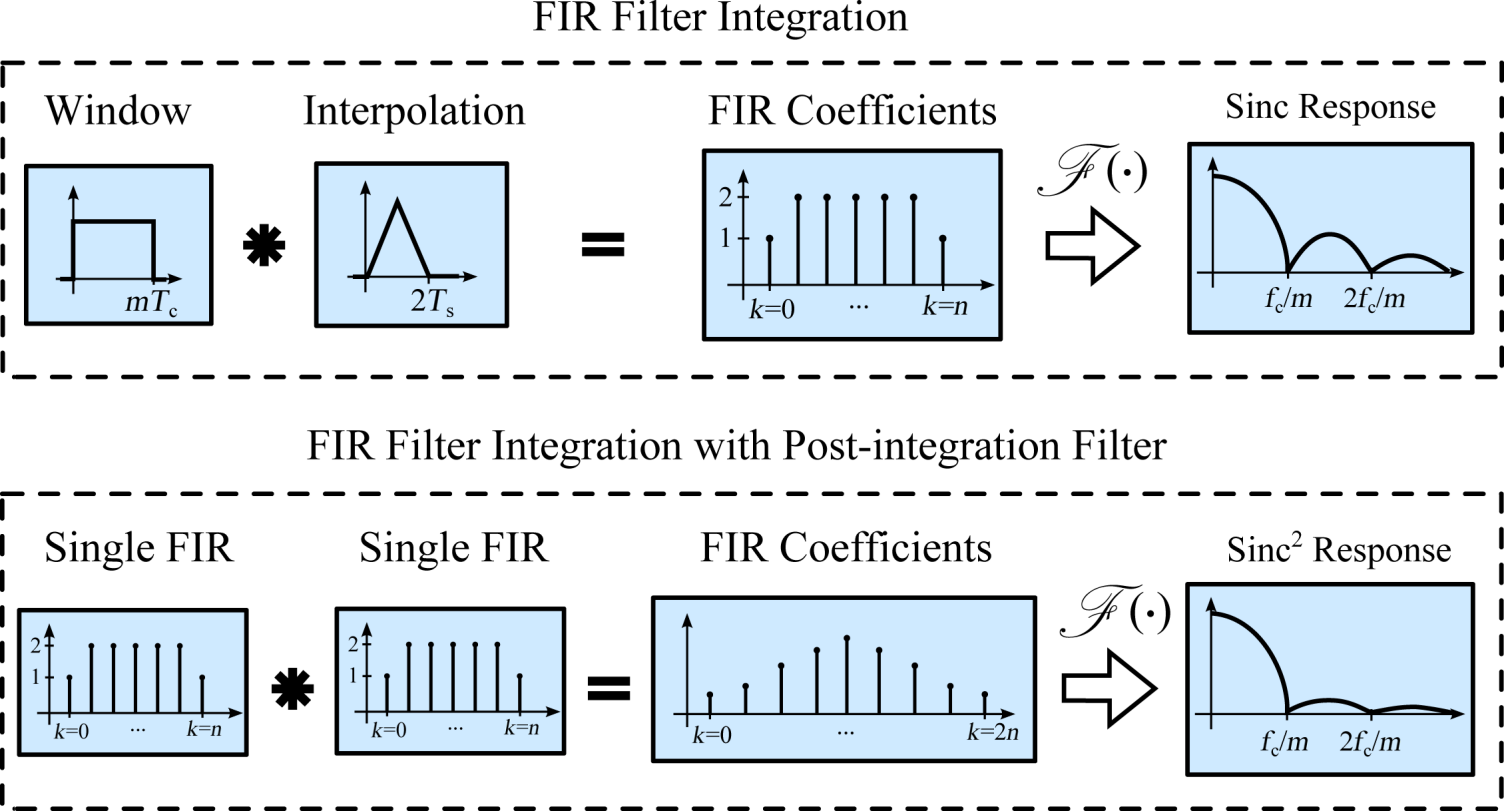
Schematic signal flow of the numerical integration scheme employed by the coherent demodulator with and without post-integration filter. Since 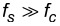, the frequency response of the interpolation filter can be neglected.

By writing out the sum of the discrete-time integral using a trapezoidal interpolation method [41], it can be seen that the full-period integration can be directly realized with an (*n* + 1)-tap FIR filter with coefficients [1, 2, 2, … 2, 1] as schematically shown in Figure 10. The impulse response of this FIR filter is naturally obtained from the convolution of the rectangular integration window of length *mT_c_* with a first-order hold element of length 2*T_s_*. This is equivalent to passing the mixed signal through a sinc filter with side-lobes located at integer multiples of *f_c_*/*m*. Since *f_s_* is much higher than *f_c_*, the frequency response of the interpolation filter can be neglected.

It is clear that if the integration window length is chosen to be integer multiples of the oscillation period, the sinc filter will have zeros at the harmonics of the oscillation frequency. If the integration period can be made infinitely precise, this approach will remove harmonics from the output. However, as *nT_s_* = *mT_c_* rarely holds, the partial integration is difficult to do precisely and the input signal may contain a DC offset, harmonics will still appear in the output of the integration method. As such, this method can be improved by employing a high-pass filter on the input and post-integration filters [43], either in the form of a direct notch filter at the second harmonic or by passing the output through another numerical integrator [41]. Intuitively, as Figure 10 illustrates, this method can be viewed as a FIR filter with a triangular impulse response obtained from the convolution of the two FIR integration filters, resulting in a sinc^2^ frequency response with significantly reduced side-lobes (for a direct comparison see Appendix B.

While simulation results show that low latency and high tracking bandwidth can be achieved for a pure sinusoid by integrating over one period *M* = 1, in order to reject white noise, multiple oscillation periods must be integrated which reduces the tracking bandwidth and increases the latency [42]. In this work, the trapezoidal numerical integration method with post-integration filters described in [42–43] is directly implemented by cascading two FIR integration filters. Alternatively, the second FIR filter can be replaced by a notch filter at the second harmonic. The computational efficiency of this method can be increased by computing the integral cumulatively, as described in [41–42].

The original work presenting the coherent demodulator integrates over a full period of the fundamental frequency to achieve the highest tracking bandwidth (corresponding to *M* = 1 and *n* = 6 in this work). However, the highest possible tracking bandwidth can be achieved by setting *n* = 3, which still guarantees that the component at 2*f_c_* is exactly canceled. A comparison of the original and half-period coherent demodulator is presented in Appendix B.

### Summary

Table 1 compares the amplitude estimation techniques discussed in this section. From the classification shown in Figure 2, methods based on rectification can only obtain amplitude estimates while methods based on mixing with an internal reference oscillator can recover both amplitude and phase. Additionally, some of these methods require precise synchronization between the sampling frequency and reference signal. In practice, this requires a single system clock for the sampling time and signal generation. While this property is not a disadvantage when using FPGA-based processing, it does affect the choice of carrier frequencies for the coherent demodulator if the integral is to be precise.

**Table 1:** Qualitative summary of amplitude estimation methods stating the tracking bandwidth tuning parameter, ability to determine a phase estimate and timing requirements.

method	tuning parameter	phase estimate	timing requirement	references

lock-in amplifier	LPF *f*_lp_	yes	no	[30–32]
HBW lock-in amplifier	LPF *f*_lp_	yes	no	[35]
Kalman filter	*Q*	yes	no	[44,47–48]
Lyapunov filter	γ	yes	no	[45–46,49]
moving average filter	# of samples	no	yes	[29,57]
mean absolute deviation	LPF *f*_lp_	no	yes	[29]
peak hold	LPF *f*_lp_	no	yes	[36,38]
peak detector	LPF *f*_lp_	no	no	[45]
coherent demodulator	# of samples	yes	yes	[40–43,65]

## Experimental evaluation

### Experimental setup

The aforementioned demodulation techniques were implemented digitally on a common DSP system (National Instruments USB-7855R with Kintex-7 70T FPGA) using dedicated LabVIEW blocks and simple scalar operations. This system was chosen due to its system-oriented graphical design approach, which makes it an accessible FPGA tool without the need for knowledge of hardware description languages.

For a fair comparison and to rule out varying amounts of quantization noise, all demodulation methods are run at a normalized sample frequency of *f_s_* = 300 kHz. However, this may not do full justice to the fastest running methods as these techniques might benefit from noise reduction due to over-sampling. Additionally, the methods requiring accurate timing will also benefit from more samples per oscillation period.

### Implementation complexity

The sample rate achieved by any FPGA implementation, irrespective of the hardware, is a function of the sequential computations which are carried out during each sample period [66]. Therefore the maximum free-running sampling rates, listed below in Table 2, are used to qualitatively compare the implementation complexities.

Due to their simple implementations, the mean absolute deviation method, the peak detector and the moving average filter achieve the highest sampling rates with the mean absolute deviation method approaching the maximum achievable rate of the FPGA system of 1 MHz. The lock-in amplifier, high-bandwidth lock-in amplifier and coherent demodulator achieve the next highest sample rates, while the Lyapunov filter and Kalman filter run at around 300 kHz. Although the Kalman filter is significantly more complex than the Lyapunov filter, the small difference of only 27 kHz can be associated with the highly hardware-optimized implementation of the Kalman filter [44,48], which does not use any continuous states or LabVIEW specific blocks.

### Tracking bandwidth

The tracking bandwidth of each demodulator is determined by measuring the amplitude tracking frequency response. This was performed using a laboratory function generator (Agilent 33521A Waveform Generator) to provide a carrier frequency of *f_c_* = 50 kHz, which is amplitude-modulated by a frequency-swept sine signal using the external modulation input. The −3 dB modulation bandwidth of the waveform generator was experimentally verified to be 103.9 kHz, surprisingly low compared to the 30 MHz generator bandwidth but large enough for the carrier frequency used in this experiment. This FM–AM concept directly reveals the low-pass filter characteristic of the demodulators and allows for a direct extraction of the −3 dB tracking bandwidth.

**Figure 11:**
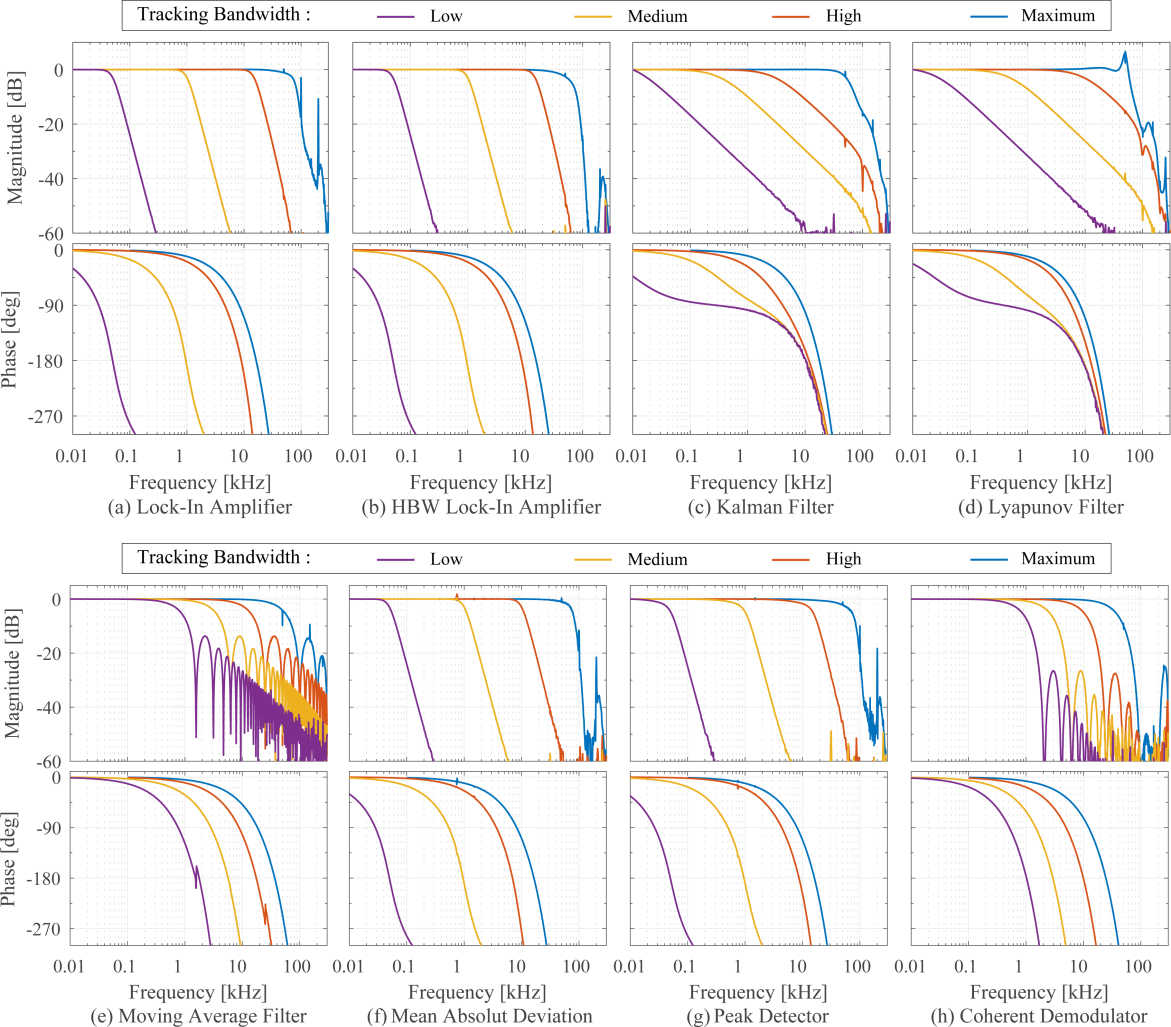
Tracking bandwidth frequency response of the demodulators showing the −3 dB tracking bandwidth and equivalent filter order for four different bandwidth settings each.

The results are presented in Figure 11 where four different tracking bandwidths are plotted. The maximum achievable tracking bandwidth for each technique is stated below in Table 2. Apart from the tracking bandwidth, the equivalent demodulator filter order (determined from the amplitude reduction per decade for the slowest bandwidth setting) can also be determined from this plot and is stated below in the Summary subsection in Table 2. However, as every demodulator operates nonlinearly, such a classification is only an approximation. As the moving average filter and coherent demodulator are effectively sinc and sinc^2^ filters, we have approximated these by fitting to the local maxima of the side-lobes. From this experiment the linear relationship between the demodulator tuning variable and resulting tracking bandwidth can be obtained, which is discussed in more detail in Appendix C.

It can be seen that the lock-in amplifier and the mean absolute deviation method followed by the peak detector achieve the highest bandwidth, however, at the expense of passing through large 2*f_c_* components, which are visible in Figure 21a,f,g. Comparing Figure 21a with Figure 21b, the elimination of the peaks due to the phase cancellation of the high-bandwidth lock-in amplifier is clearly visible.

The Kalman filter achieves a maximum bandwidth of around 50 kHz, which corresponds to tracking within one cycle of the carrier signal without any distortion. However, the Lyapunov filter achieves a slightly higher maximum bandwidth of around 59 kHz but at the expense of peaking at the carrier frequency. This fact is due to the filter recovering the sum of the carrier and the modulating frequency, hence a gain of 2 (6 dB) is measured at 50 kHz.

The FIR filters in the coherent demodulator and the moving average filter implementation cause a characteristic sinc/sinc^2^ frequency response, mathematically originating from the Fourier transform of the integration window. The maximum tracking bandwidth of the coherent demodulator is 39.0 kHz without post-integration filter and 28.6 kHz with post-integration filter. These values correlate with the time-domain simulation in [42], which show a convergence after around 1–2 cycles.

### Sensitivity to other frequency components

**Figure 12:**
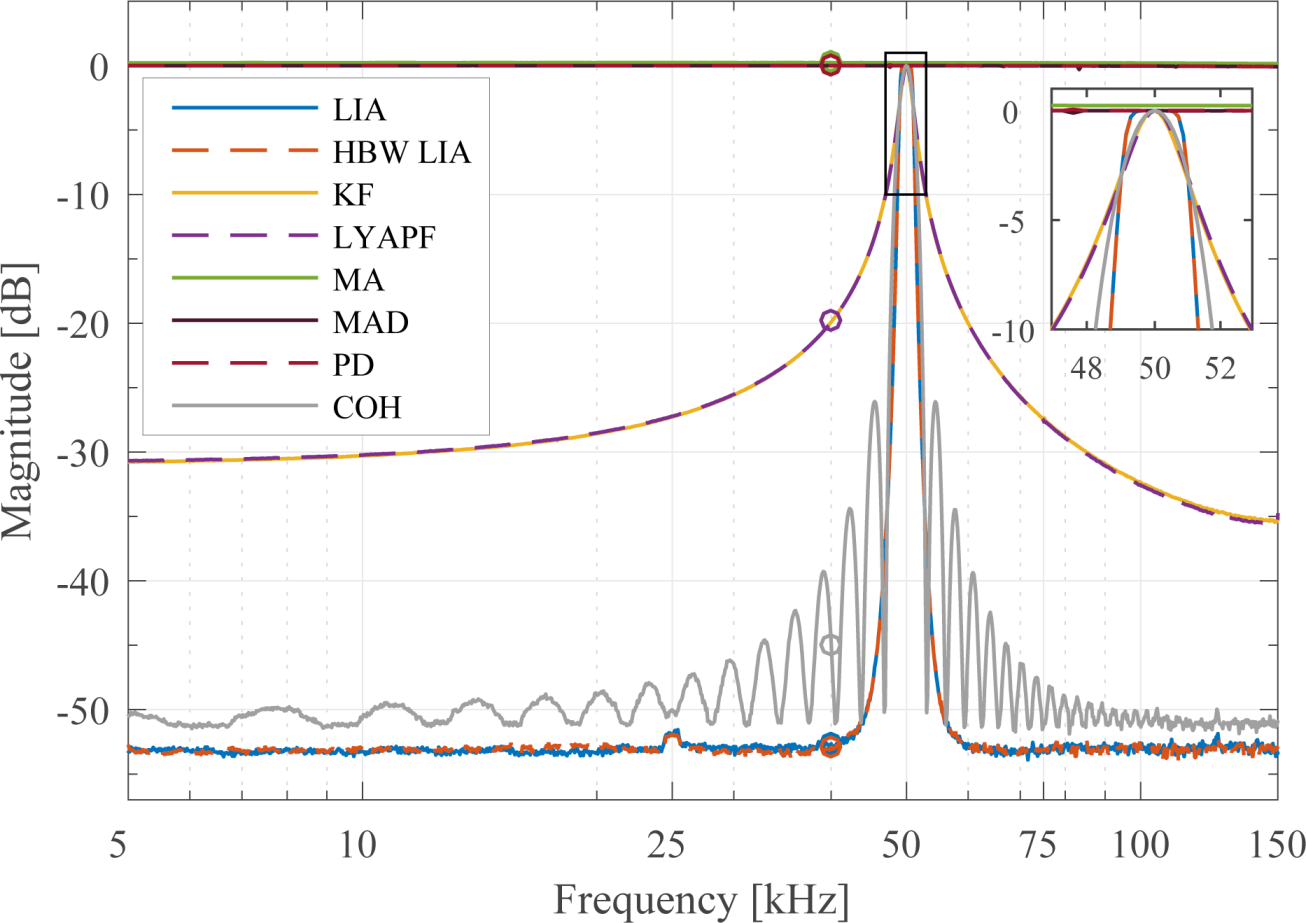
Off-mode rejection of the demodulators for a carrier frequency of *f_c_* = 50 kHz and a tracking bandwidth of 1 kHz. The zoom box highlights the intersection at the points (50 ± 1 kHz, −3 dB) and the different filter shapes at the modeled carrier frequency.

In order to determine the sensitivity to other frequency components present in the signal to be demodulated, a frequency sweep on the carrier signal is performed while the demodulators (where possible) are set to a specific frequency (*f_c_* = 50 kHz) and the demodulation bandwidth is set to a fixed value of 1 kHz using the relationships plotted in Appendix C. The resulting plot in Figure 12 shows the attenuation of frequencies other than the modeled carrier frequency and is therefore termed off-mode rejection (OMR). As a quantitative comparison parameter, the OMR is calculated as the gain difference at the modeled frequency (0 dB) and at 40 kHz as highlighted in Figure 12 and stated below in Table 2. In this experiment, the noise floor far away from the modeled frequency is limited by the residual DC-offset caused by the finite quantization of the digital-to-analog converter (DAC) of the amplitude estimator. However, as all methods are measured with the same hardware, the relative difference is a good indication of maximum achievable off-mode rejection values.

The rectification methods that do not make any assumption on the carrier frequency such as the moving average (MA) filter, mean absolute deviation (MAD) method and peak detector (PD) show a constant gain across all frequencies. In other words, these methods are very sensitive to additional frequency components in the signal as they recover all frequencies equally and are hence impractical for multifrequency AFM.

On the other hand, the lock-in amplifier (LIA) and the high-bandwidth lock-in amplifier (HBW LIA) yield the best off-mode rejection of around −52.0 dB owing to the fourth-order Butterworth low-pass filters employed. This result emphasizes the fact that these methods are very insensitive to additional frequency components in the signal and should be used when maximum suppression of these components is of priority.

The Lyapunov filter (LYAPF) and the Kalman filter (KF) yield an off-mode rejection of around −20 dB, significantly lower than the two lock-in amplifier implementations. This fact is due to the equivalent first order response of these filters as shown in Figure 11c,d and stated below in Table 2.

The equivalent order of the coherent demodulator (COH) follows from the envelope of the sinc^2^ frequency response. It can be seen that the off-mode rejection is maximized at frequencies corresponding to the zeros of the sinc^2^ function. This in turn means that broadband white noise or noise at frequencies other than at these zeros cannot be sufficiently suppressed. This is in contrast to the lock-in amplifier and high-bandwidth lock-in amplifier which show a constant large off-mode rejection away from the carrier frequency.

**Figure 13:**
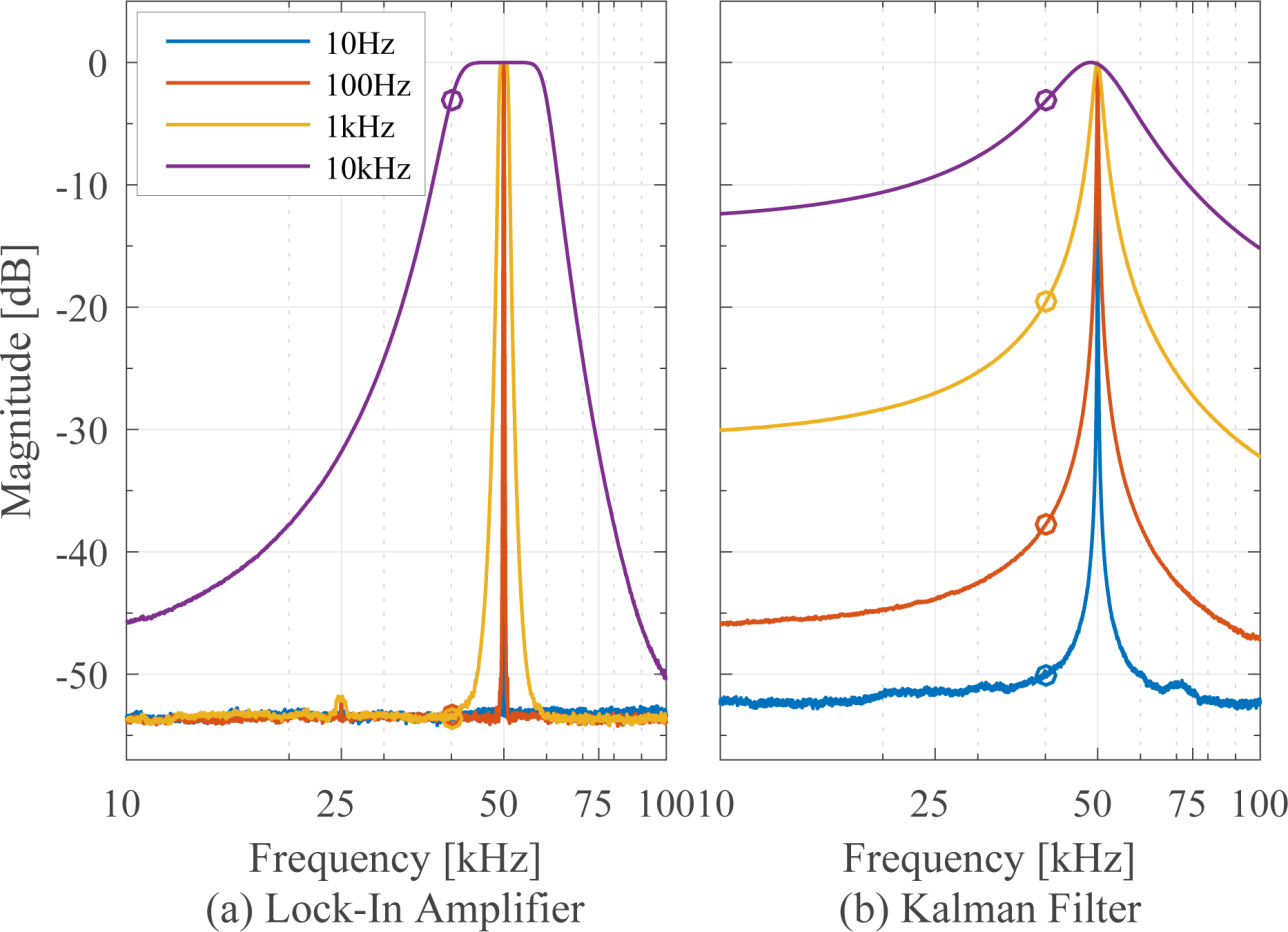
Off-mode rejection of (a) fourth-order lock-in amplifier and (b) first-order Kalman filter for a carrier frequency of *f_c_* = 50 kHz at 10 Hz, 100 Hz, 1 kHz, and 10 kHz tracking bandwidth.

The off-mode rejection of the Kalman filter and Lyapunov filter can be significantly improved by lowering the tracking bandwidth as shown in Figure 13. In order to achieve a rejection of greater than −40 dB, the bandwidth must be reduced to 100 Hz. On the other hand, the lock-in amplifier only significantly loses its off-mode rejection property at large tracking bandwidths of around 10 kHz. For these large tracking bandwidths, the Butterworth nature of the post-mixing low-pass filters is clearly evident in Figure 13a. The flat region around the modeled frequency where the amplitude is within −3 dB corresponds to twice the tracking bandwidth. The tuning for the Kalman filter is described in Appendix C.

### Noise evaluation

**Figure 14:**
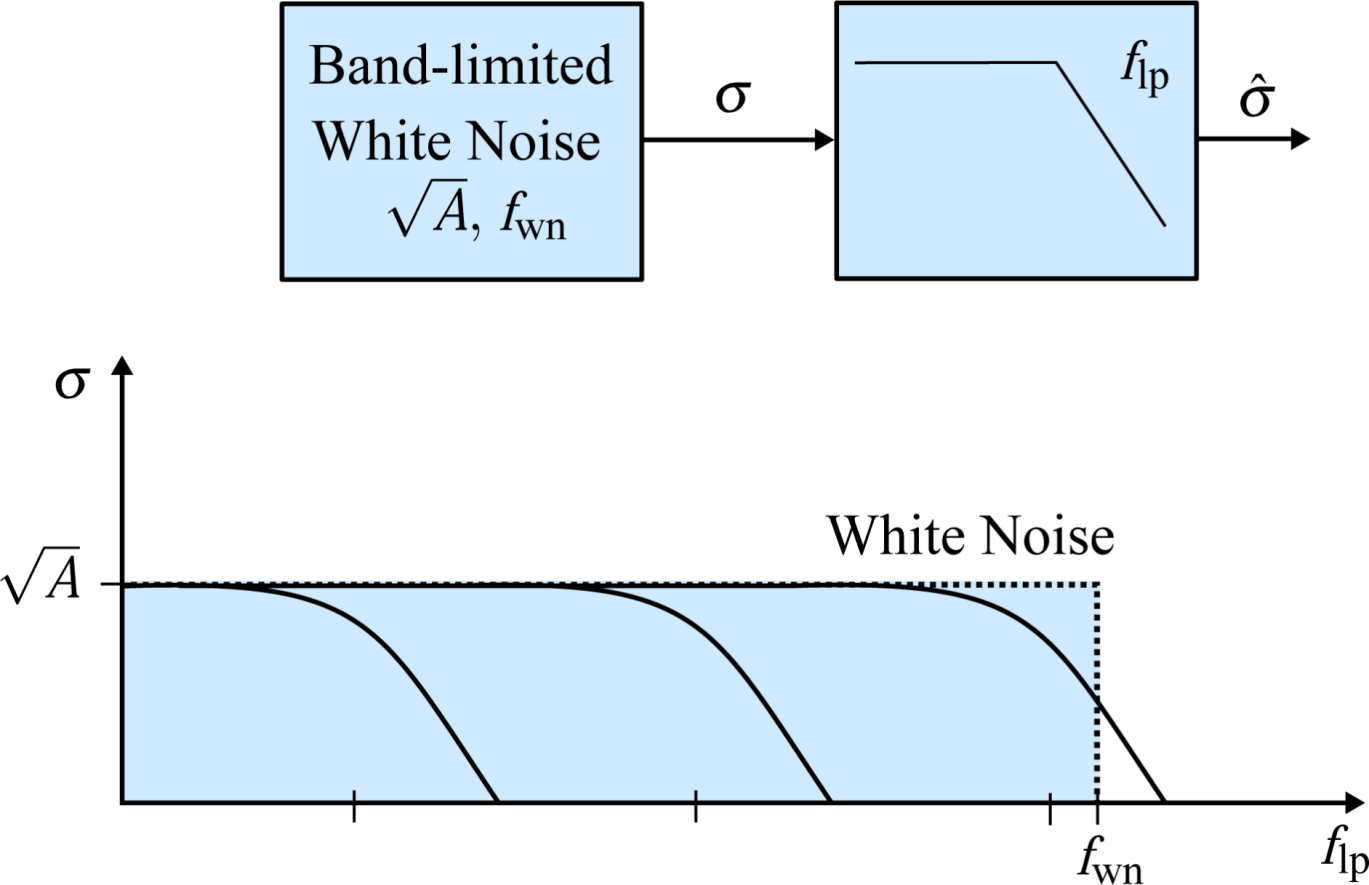
Schematic block diagram of the reference experiment of filtering a band-limited white noise process with a variable cut-off frequency low-pass filter and its schematic representation in the frequency domain.

In order to determine the noise performance, the RMS noise of the amplitude estimate is evaluated as a function of the tracking bandwidth. The responses are compared against the theoretical and experimental response of an “ideal demodulator” represented by a low-pass filtered white noise process. A schematic block diagram of the reference experiment is shown in Figure 14. The band-limited white noise process can be described by a constant power spectral density within the bandwidth, i.e. [67],

[18]
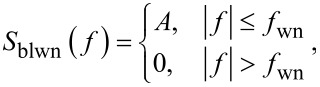


where *f*_wn_ is the white noise bandwidth in Hz and *A* is the power spectral density in *V*^2^/Hz. The RMS noise value σ can be obtained by calculating the total integrated noise (TIN) of the output of a system *G* driven by a white noise input which is given by [67]

[19]
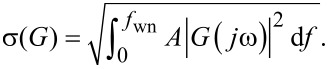


For the perfect band-limited system (Equation 18), *G* = 1 and Equation 19 simplifies to

[20]
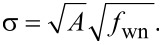


The reference curves obtained from this experiment (see Appendix D for details) can be considered as “ideal demodulators” and are compared to the modulated white noise experiment that is schematically shown in Figure 14.

**Figure 15:**
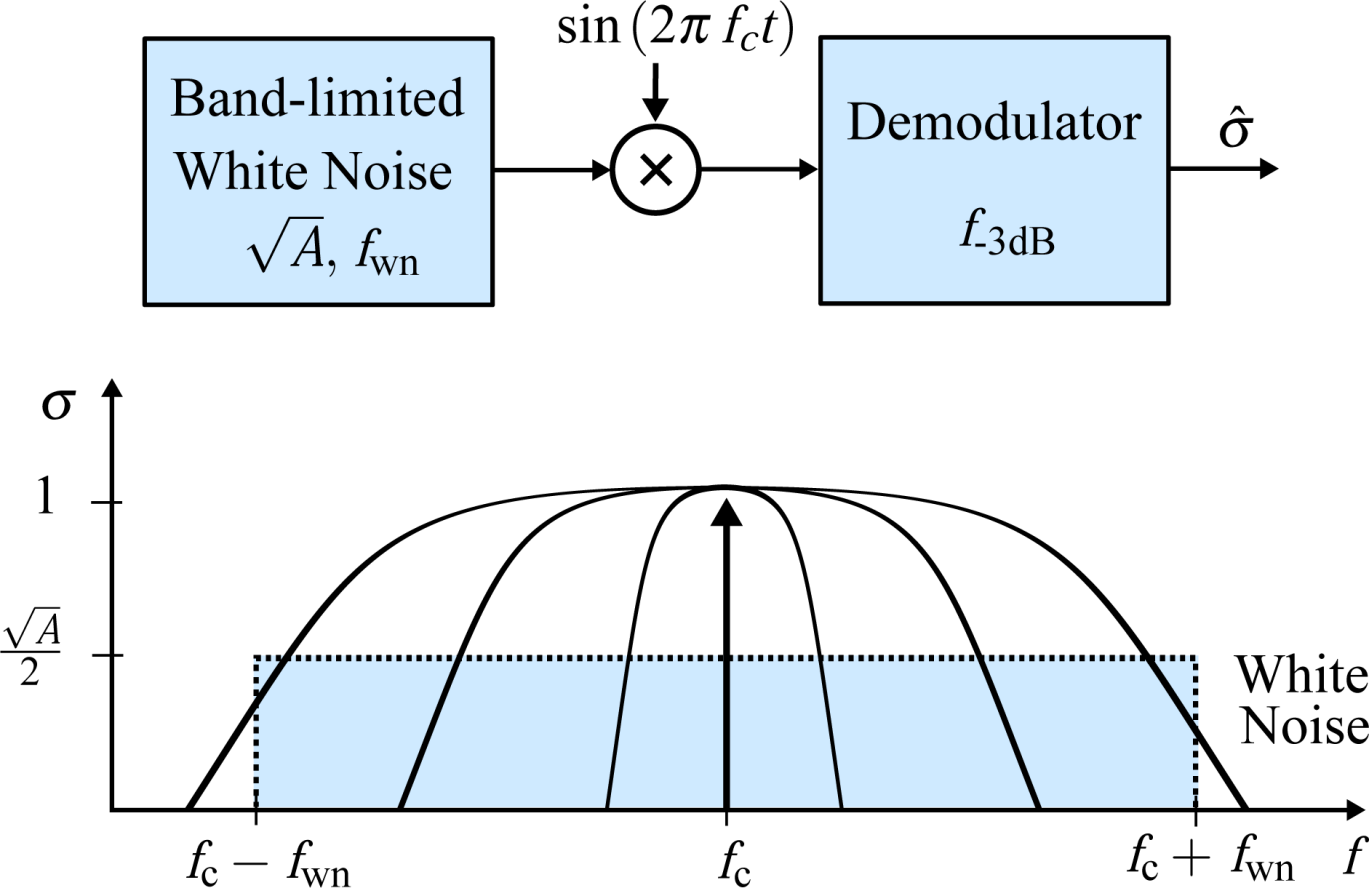
Schematic block diagram of bandwidth-vs-noise experiment of recovering an amplitude-modulated band-limited white noise process with demodulators with variable tracking bandwidths and its schematic representation in the frequency domain.

In this experiment, a laboratory function generator (Agilent 33521A Waveform Generator) providing a 40 kHz bandwidth-limited white noise signal with an amplitude of 10 mV_rms_ is first measured directly through a second-order variable cut-off frequency low-pass filter (Stanford Research SR560 Low Noise Preamplifier). The acquisition front end of a micro system analyzer (Polytec MSA-050-3D) is used to capture the time-domain data sampled at *f_s_* = 2.56 MHz for *T* = 13.11 s. The TIN is obtained by integrating the noise density estimate from DC to *f_s_*/2 using Welch’s method with 16 averages. Subsequently, each demodulator is subjected to amplitude-modulated white noise as shown in Figure 15 with a carrier frequency of 50 kHz and the demodulated amplitude is recorded for several tracking bandwidths in the same manner.

**Figure 16:**
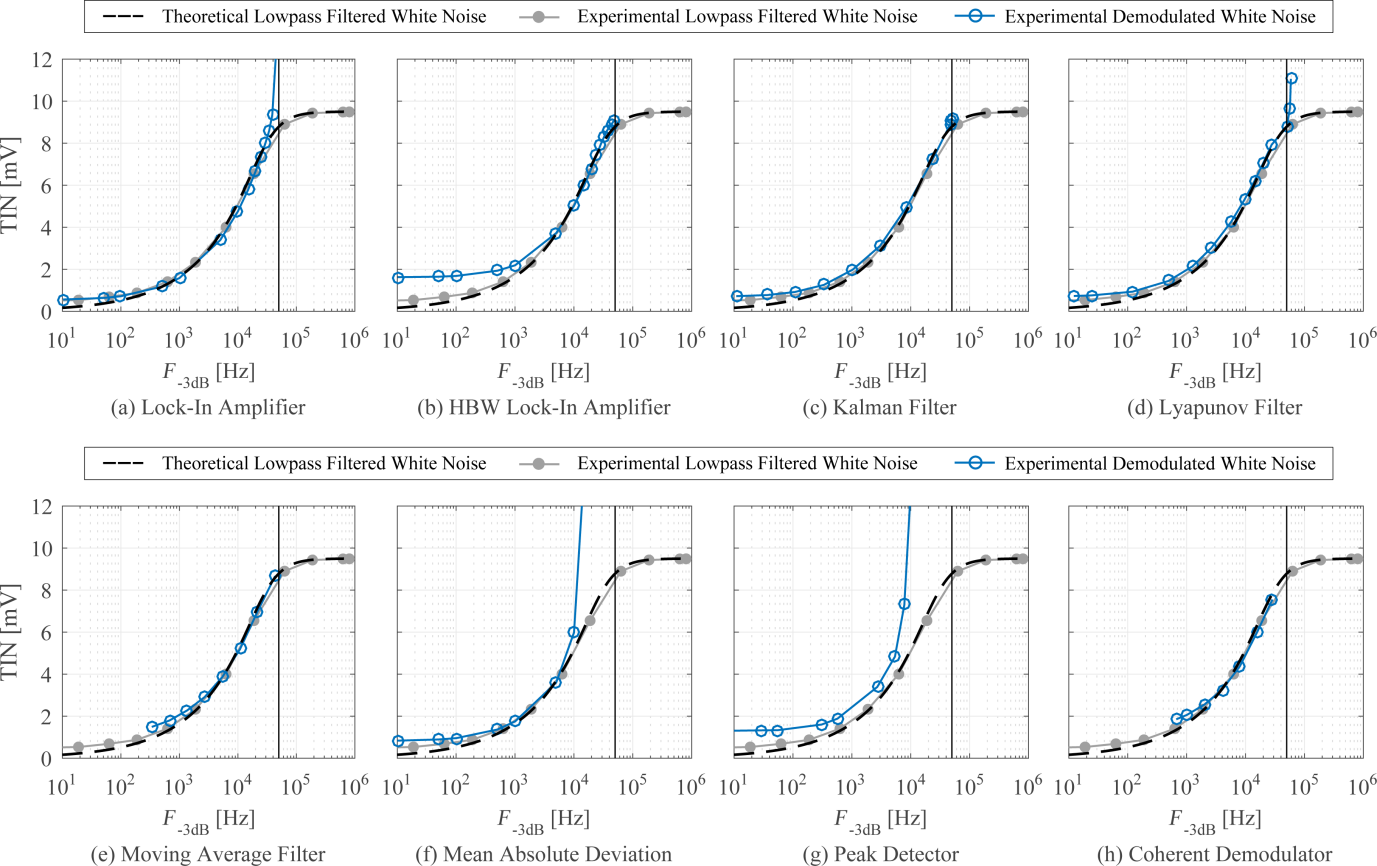
Tracking bandwidth vs total integrated noise (TIN) for each demodulator (blue circles) and for a low-pass filtered white noise process (gray dots) and its analytical expression (dashed black line) as reference. The vertical line indicates the location of the carrier frequency *f_c_* = 50 kHz.

For each demodulator, the results are shown in Figure 16. It can be seen that the lock-in amplifier follows the trend of the reference filtered white noise process for low tracking bandwidths but exhibits an exponentially growing TIN when the tracking bandwidth approaches the carrier frequency. This fact is due to the increasing 2*f_c_* component in the amplitude estimate due to inadequate filtering of the mixing product. On the contrary, the high-bandwidth lock-in amplifier does not show this increase owing to the phase cancellation employed. However, the addition of the phase-shifted mixing products increases the noise for lower tracking bandwidths.

The Kalman filter and the Lyapunov filter show an equal trend without any noise increase until the bandwidth reaches the carrier frequency. The Kalman filter never crosses this point. In contrast, the succeeding nonlinearity displayed by the Lyapunov filter effectively reduces its useful bandwidth to that of the Kalman filter. The ability of the moving average filter and the coherent demodulator to reach the lower tracking bandwidth frequency range is dictated by the highest-order FIR filter that can be implemented on the FPGA. For the moving average filter this is *n* = 384 and for the coherent demodulator with post-integration filters this limit is *n* = 144 for the LabView hardware used in this work. Lastly, the mean absolute deviation method and the peak detector are constrained by inadequate filtering of the mixing products arising from the absolute value operation, which significantly limits their practical bandwidth.

At frequencies approaching DC, all methods, including the low-pass filtered white noise process, approach a constant value due to digital noise, residual DC-offsets and 1/*f* noise in the signal. This experiment highlights that amplitude noise needs to be taken into account when stating the maximum tracking bandwidth of demodulation methods.

### Summary

Table 2 summarizes the results of the amplitude estimation techniques evaluated in this section. The results show that several demodulation methods are able to obtain amplitude estimates in a single cycle, corresponding to a maximum tracking bandwidth *f*_−3dB_ = *f_c_*. However, this figure of merit needs to be assessed with caution as it does not reflect the noise present in the amplitude estimate due to insufficient filtering of mixing products. For instance, the lock-in amplifier can only be used up to 38 kHz (compared to 70 kHz as stated in Table 2) before the harmonic distortion makes this demodulator unusable. While the high-bandwidth lock-in amplifier eliminates this problem, the addition of orthogonal sinusoids increases the noise for low bandwidths. In contrast, the Kalman filter and Lyapunov filter, despite being of low order, show excellent noise performance over the entire bandwidth of interest. The feasible tracking bandwidth range for each demodulator can be read from Figure 16. The sensitivity to other frequency components is assessed by the off-mode rejection experiment, which measures the ability to reject white noise or other deterministic frequency components. Higher off-mode rejection is achieved by increasing the equivalent order of the demodulator.

**Table 2:** Maximum free-running sample frequency *f*_s_, maximum tracking bandwidth *f*_−3dB_, equivalent order and off-mode rejection (OMR) at 1 kHz tracking bandwidth of each demodulation technique.

method	max. *f*_s_ [kHz]	max. *f*_−3dB_ [kHz]	order	OMR [dB]

lock-in amplifier	431	70.0	4	53
HBW lock-in amplifier	417	52.6	4	53
Kalman filter	300	50.5	1	20
Lyapunov filter	327	58.7	1	20
moving average	580	43.3	1	0
mean absolute deviation	977	70.9	4	0
peak hold	580	21.7	4	0
peak detector	800	58.5	4	0
coherent demodulator	362	28.6	2	45

## AFM imaging

In order to demonstrate the effect of insufficient demodulator bandwidth, a high-speed tapping-mode AFM experiment is conducted with a NT-MDT NTEGRA AFM equipped with a Bruker DMASP piezoelectrically actuated cantilever. Imaging was performed in constant-height mode to circumvent the common *z*-axis actuator bandwidth limitation. Since the *z*-axis controller bandwidth is reduced to the point where the sample features entirely appear in the amplitude error image, any imaging artifacts are either due to insufficient demodulator or cantilever bandwidth.

**Figure 17:**
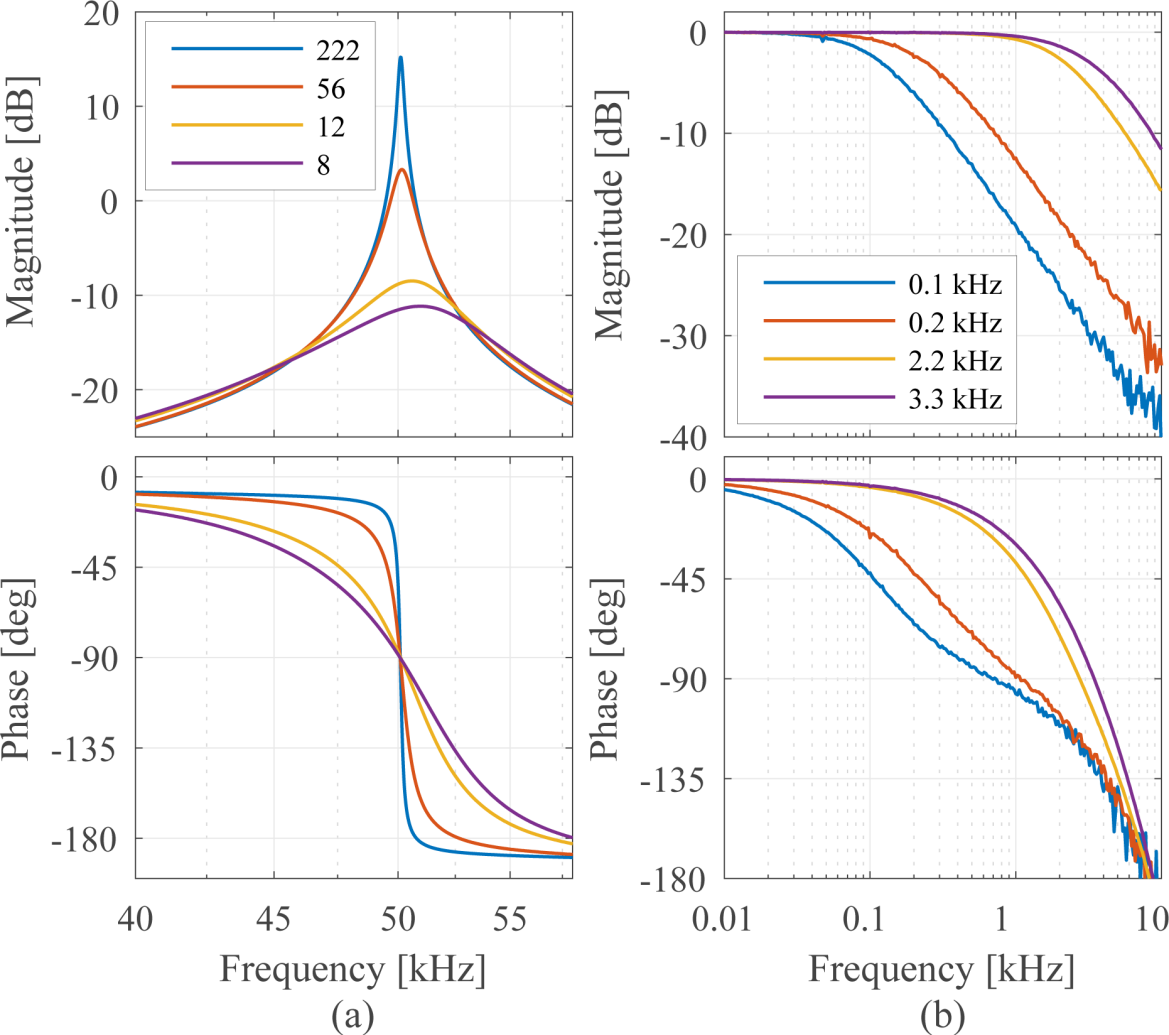
(a) Frequency response of the DMASP cantilever in open-loop (blue) and for various quality factor controller gains to reduce the quality factor as stated in the legend. (b) Tracking bandwidths of the DMASP cantilever determined via drive amplitude modulation for the frequency responses shown in (a) and color-coded accordingly.

In order to render the demodulator the bottleneck, the fundamental resonance at *f*_1_ ≈ 50 kHz of the DMASP cantilever is heavily damped with model-based quality factor control [68]. The frequency responses from the cantilever actuation to tip displacement for various quality factor controller gains are shown in Figure 17 along side the corresponding tracking bandwidths obtained from drive amplitude modulation. Due to the integrated actuation of the cantilever, the control method achieved a quality factor as low as *Q*_1_ = 8, resulting in a tracking bandwidth of 3.3 kHz, adequately matching the first-order approximation *f*_1_/(2*Q*_1_) [36].

AFM images of a calibration grating (NT-MDT TGZ3) with periodic features of height *h* ≈ 500 nm were obtained at a speed of 627 μm/s and 1.25 mm/s. Because the scanner rate of the AFM is limited to 31.37 Hz, the scan areas for the two different speeds are, respectively, 10 μm × 10 μm and 20 μm × 20 μm. The image areas have been cropped to approximately the same region for a better comparison.

**Figure 18:**
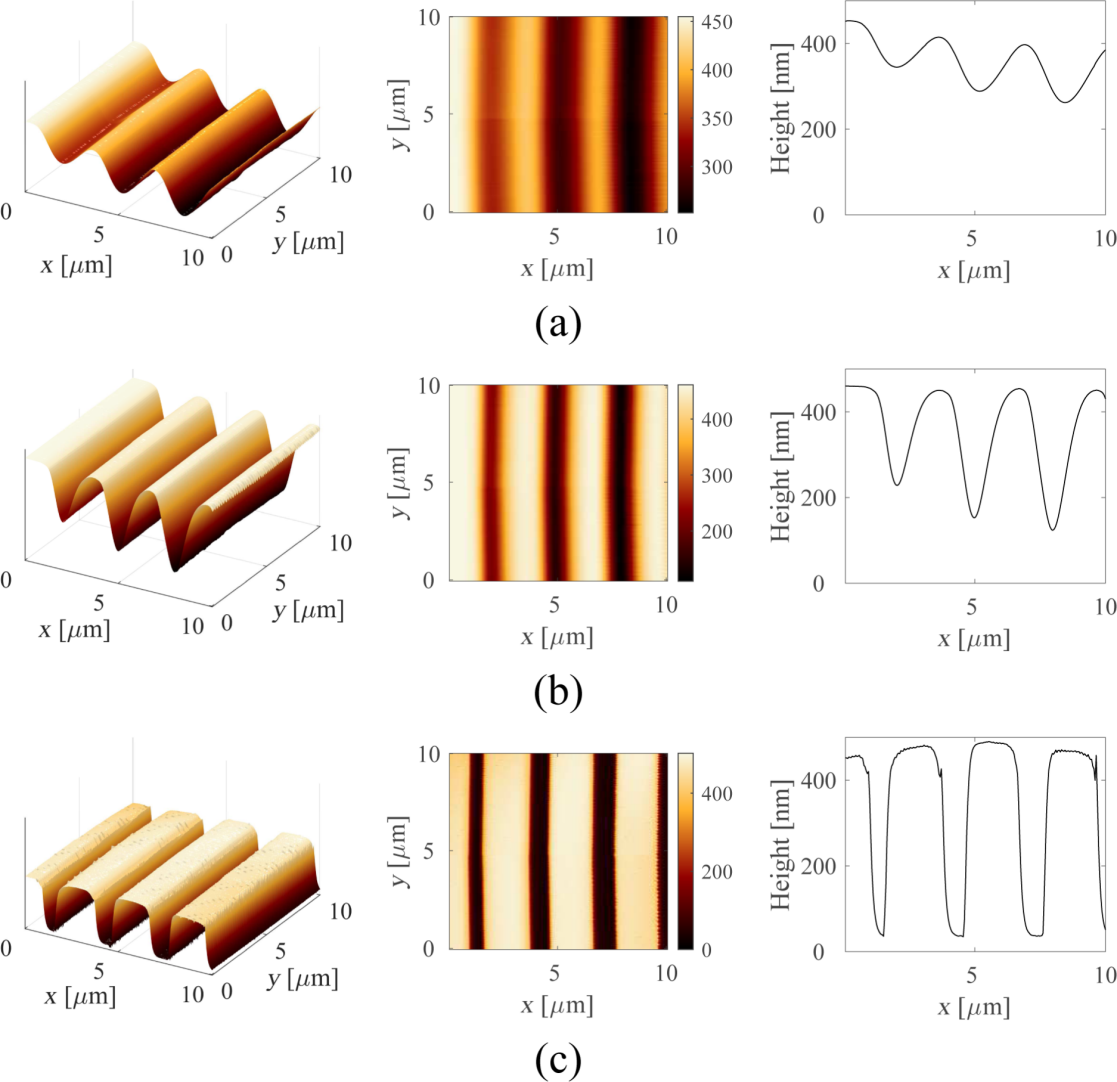
3D image, 2D image and cross section of amplitude estimates obtained from (a) lock-in amplifier with *f*_lp_ = 100 Hz, (b) lock-in amplifier with *f*_lp_ = 200 Hz, and (c) Lyapunov filter with γ = 60000 at an imaging speed of 627.45 μm/s. The scanning direction is along the positive *x*- and *y*-axes.

**Figure 19:**
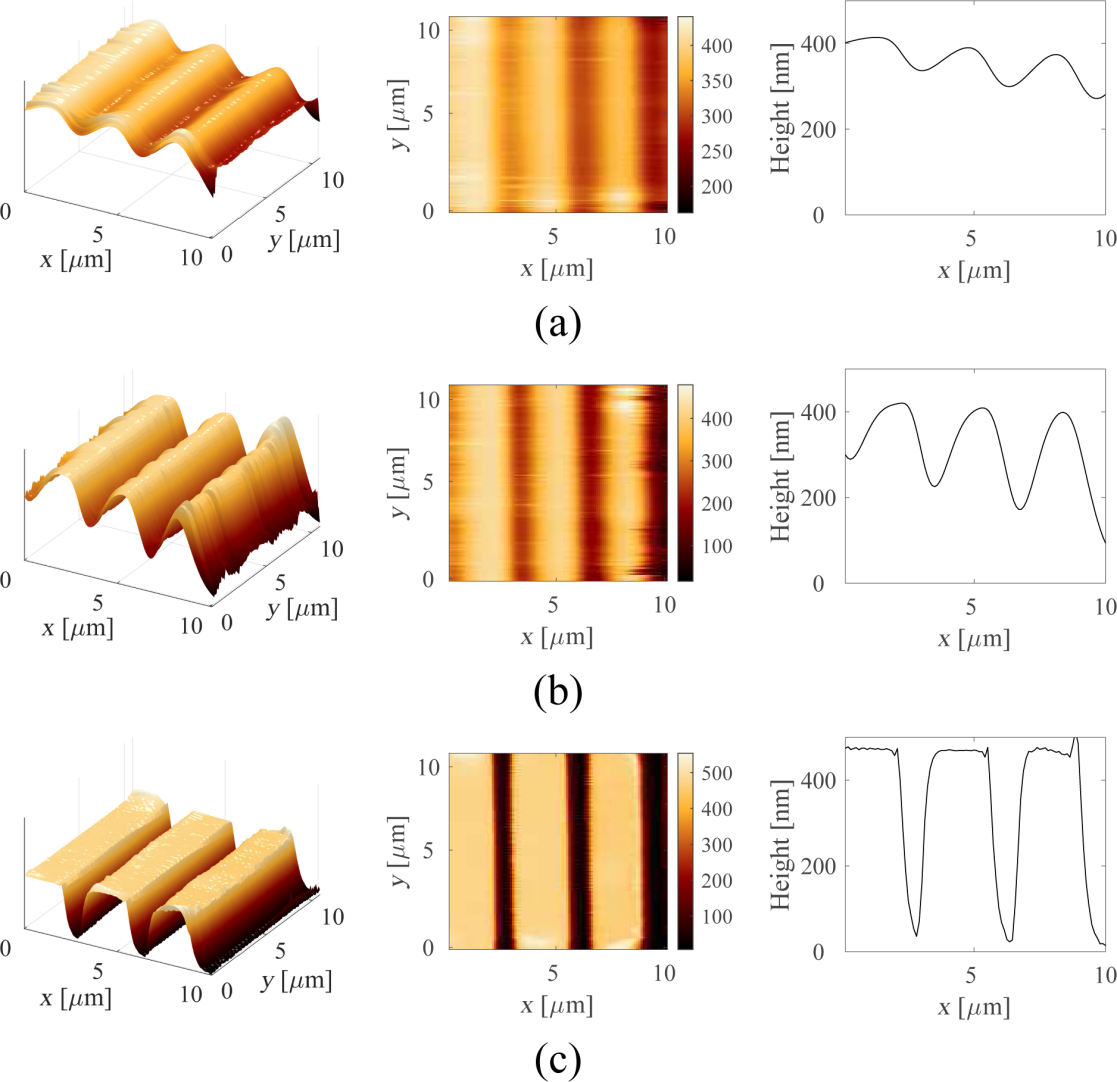
3D image, 2D image and cross section of amplitude estimates obtained from (a) lock-in amplifier with *f*_lp_ = 200 Hz, (b) lock-in amplifier with *f*_lp_ = 300 Hz, and (c) Kalman filter with *Q* = 0.004, *R* = 2 at an imaging speed of 1.25 mm/s. The scanning direction is along the positive *x*- and *y*-axes.

The high-speed constant height imaging with the lock-in amplifier, Lyapunov filter and Kalman filter are presented in Figure 18 and Figure 19. Each row corresponds to the same demodulator bandwidth. It can be seen that for small demodulator bandwidths, the sample features are not accurately tracked (first two rows of each figure). By setting a larger demodulator bandwidth, the sharp sample features are properly tracked, which is clearly evident in the cross-section plots.

Note that the purpose of the AFM images is to emphasize the need for a fast demodulator bandwidth when all other bandwidth limiting components in the AFM loop are eliminated. Therefore, the authors perform imaging in constant-height mode, which entirely removes the *z*-axis controller and actuator limitation. Due to the 3.3 kHz cantilever bandwidth, the full potential of the fastest methods cannot be utilized and hence the AFM images themselves cannot serve as a means of differentiating between these methods.

## Conclusion

This article provides an experimental comparison of the performance of conventional and novel digital demodulation techniques over their entire tracking bandwidth. The techniques include mixing-based methods namely the lock-in amplifier, high-bandwidth lock-in amplifier, coherent demodulator, Kalman filter, and Lyapunov filter, as well as rectification-based methods in the form of a moving average filter, mean absolute deviation computation and peak detection. The performance metrics considered were the tracking bandwidth, implementation complexity, sensitivity to other frequency components and tracking bandwidth vs noise performance.

The 2*f_c_* component naturally arises in demodulation schemes employing mixing, which will distort the output if not adequately filtered. The lock-in amplifier relies on general low-pass filters to attenuate these mixing products, limiting the maximum achievable tracking bandwidth. While the high-bandwidth lock-in amplifier eliminates the 2*f_c_* component via phase cancellation, it introduces additional noise at low frequencies due to the summation of the phase-shifted signals. The coherent demodulator, being an all-digital lock-in amplifier implementation, eliminates the mixing products by performing a precise numerical integration over a fixed-length time window. While this approach is able to achieve a high tracking bandwidth with minimal latency for short integration windows, a high sample to carrier frequency ratio is crucial for a high-performance implementation. The Kalman filter and the Lyapunov filter on the other hand employ internal feedback of the estimated states to reject the mixing products, which allows them to maximize the tracking bandwidth without introducing additional noise in the amplitude estimate. If maximum suppression of any signal away from the carrier frequency is the priority, the lock-in amplifier can still be regarded as the method of choice as it shows large off-mode rejection and the lowest noise at low tracking bandwidths.

Among the rectification-based methods, the RMS-to-DC conversion methods (mean absolute deviation and moving average filter) have the lowest implementation complexity. Due to their inability to reject unwanted frequency components they can only be used at small tracking bandwidths. Ando’s peak hold method requires accurate timing within the digital implementation and a high sample to carrier frequency ratio to detect the zero-crossing accurately. A modified peak hold method (peak detector) alleviates the sample rate requirement, but insufficient filtering of the absolute value distortion requires low tracking bandwidths.

The above discussion highlights that there exist multiple trade-offs. Although there are many possible application goals, three of the most common are listed below along with the recommended demodulator.

1. Maximum bandwidth: The Kalman filter provides maximum tracking bandwidth without introducing excess noise or distortion. However, the Lyapunov filter performs comparably but is significantly simpler to implement.
2. Maximum noise suppression: The lock-in amplifier provides maximum off-mode rejection when the tracking bandwidth is low enough to avoid ripple.
3. Minimum implementation complexity: The RMS-to-DC conversion methods are simplest to implement but are very sensitive to other frequency components as they do not provide any off-mode rejection.

## Appendix

### A Kalman filter equations

The recursive implementation of the Kalman filter equations follows [62–63] by iterating between the prediction step

[21]
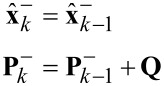


and the measurement update step by calculating the Kalman gain **k***_k_*

[22]
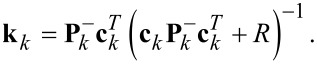


The estimated states must then be corrected

[23]
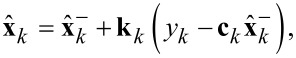


and the covariance matrix can be updated with

[24]
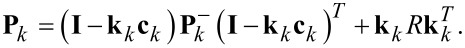


**Figure 20:**
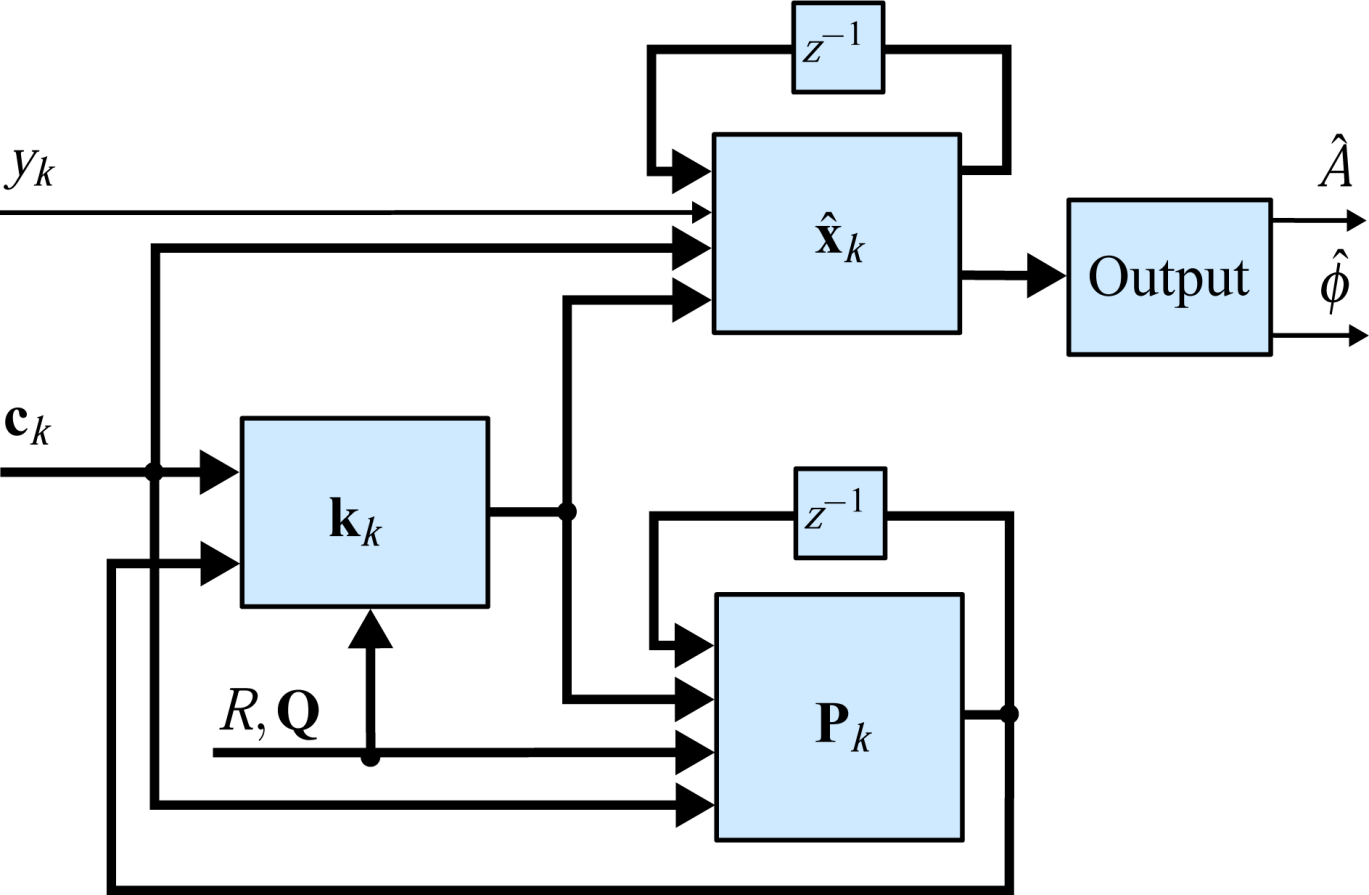
Functional block diagram of the Kalman filter implementation. Thick lines indicate vector-valued signal paths and thin lines indicate scalar signal paths.

The main computations in Equations 22–24 are graphically represented by the block diagram shown in Figure 20. Due to the time-varying system representation, the calculations in the prediction steps (Equation 21) are heavily simplified, benefiting a high-bandwidth FPGA implementation that can be realized with scalar operations [48].

### B Comparison of coherent demodulator methods

**Figure 21:**
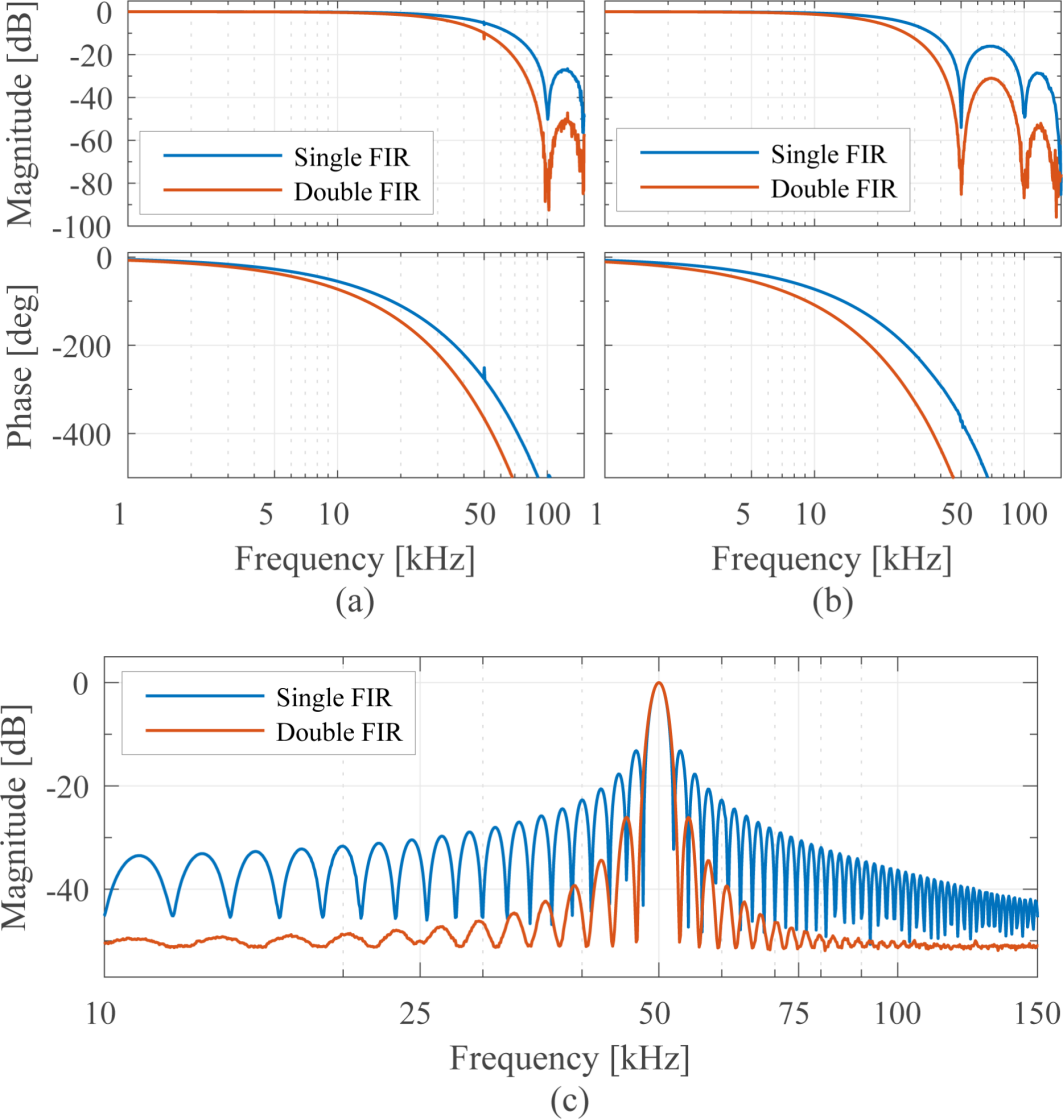
Maximum tracking bandwidth of (a) coherent demodulator (*n* = 6) and (b) half-period coherent demodulator (*n* = 3) with single FIR integration filter (blue) and FIR integration filter with post-integration filter (red). (c) Off-mode rejection at 1 kHz tracking bandwidth of coherent demodulator with single FIR integration filter (blue) and FIR integration filter with post-integration filter (red) using *f_s_* = 300 kHz, *f_c_* = 50 kHz.

Figure 21 shows a direct comparison of the coherent demodulator and half-period coherent demodulator using a single FIR integration filter (Single FIR) and with an additional post-integration filter (Double FIR). A higher attenuation at integer multiples of the carrier frequency due to the sinc^2^ frequency response of the latter is responsible for the reduction of harmonics in the output of the demodulator. Notice, that this approach naturally comes at the expense of tracking bandwidth as is visible in the magnitude response in Figure 21a. For the half-period coherent demodulator (*n* = 3), the single FIR integration filter approach yields a −3 dB tracking bandwidth of 39.0 kHz, the addition of the post-integration filter reduces this bandwidth to 28.6 kHz. For the full-period coherent demodulator (*n* = 6), the single FIR integration filter approach yields a −3 dB tracking bandwidth of 21.6 kHz, the addition of the post-integration filter reduces this bandwidth to 15.6 kHz. The increased latency is also clearly visible from the phase responses in Figure 21a,b. On the other hand, for a fixed tracking bandwidth of 1 kHz, the addition of the post-integration FIR filter improves the off-mode rejection drastically as is visible in Figure 21c because of the faster roll-off of the equivalent sinc^2^ filter.

### C Demodulator tuning

**Figure 22:**
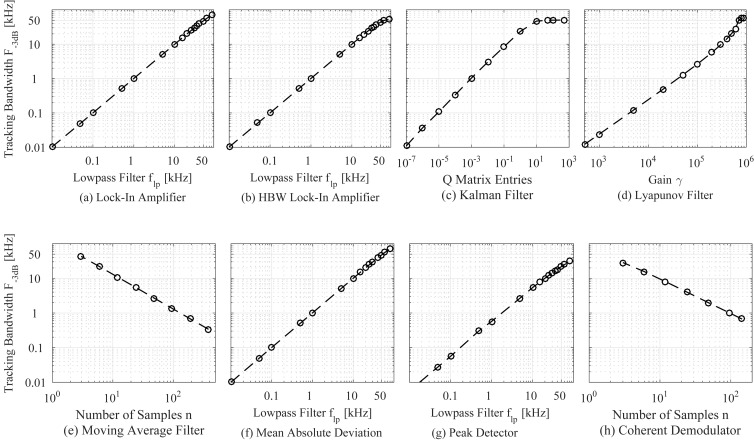
Relationship between demodulator tuning variable and achievable tracking bandwidth.

By plotting the tuning parameter against the experimentally determined tracking bandwidth in Figure 22, the region of linear relationship is determined. For the lock-in amplifier, high-bandwidth lock-in amplifier, mean absolute deviation method, and peak detector the tuning variable is the low-pass filter (LPF) cut-off frequency *f*_lp_. With a known measurement noise covariance *R*, the Kalman filter is tuned based on the assumed covariance *Q* and the Lyapunov filter can be tuned by setting the integrator gain γ. The moving average filter and the coherent demodulator tuning is achieved by setting the amount of samples per integration window *n*.

The methods achieve a near perfect linear relationship across the entire bandwidths tested on a double logarithmic scale. At the upper range of the tracking bandwidths, the relationship function for the Kalman filter flattens out revealing the proximity to the bandwidth limitation. Also noticeable is a slight deviation from the linear trend for the Lyapunov filter associated with the peaking at the carrier frequency.

Note, that this specific plot is only accurate for the particular hardware and sample frequency chosen in this work. However, it is useful in determining a particular tuning setting necessary for a given tracking bandwidth for each demodulator. For example, a 1 kHz tracking bandwidth for the Kalman filter is achieved by *Q* = diag(10^−3^, 10^−3^).

### D Low-pass filtered white noise

Substituting a first-order low-pass filter system with cut-off frequency *f*_lp_

[25]
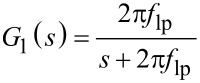


into the general expression for low-pass filtered white noise (Equation 19) yields

[26]
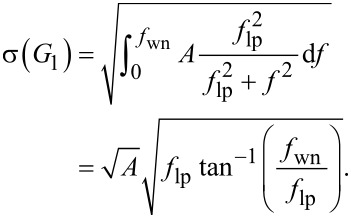


If the white noise bandwidth is much larger than the cut-off frequency 
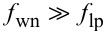
, Equation 26 can be simplified to

[27]
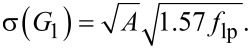


Similarly, for a second-order low-pass filter system of the form

[28]
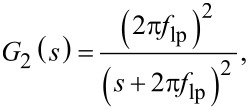


the total integrated noise evaluates to

[29]
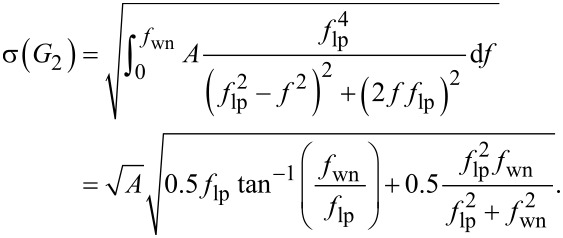


If the white noise bandwidth is much larger than the cut-off frequency 
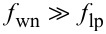
, Equation 29 simplifies to

[30]
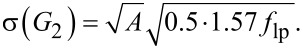


**Figure 23:**
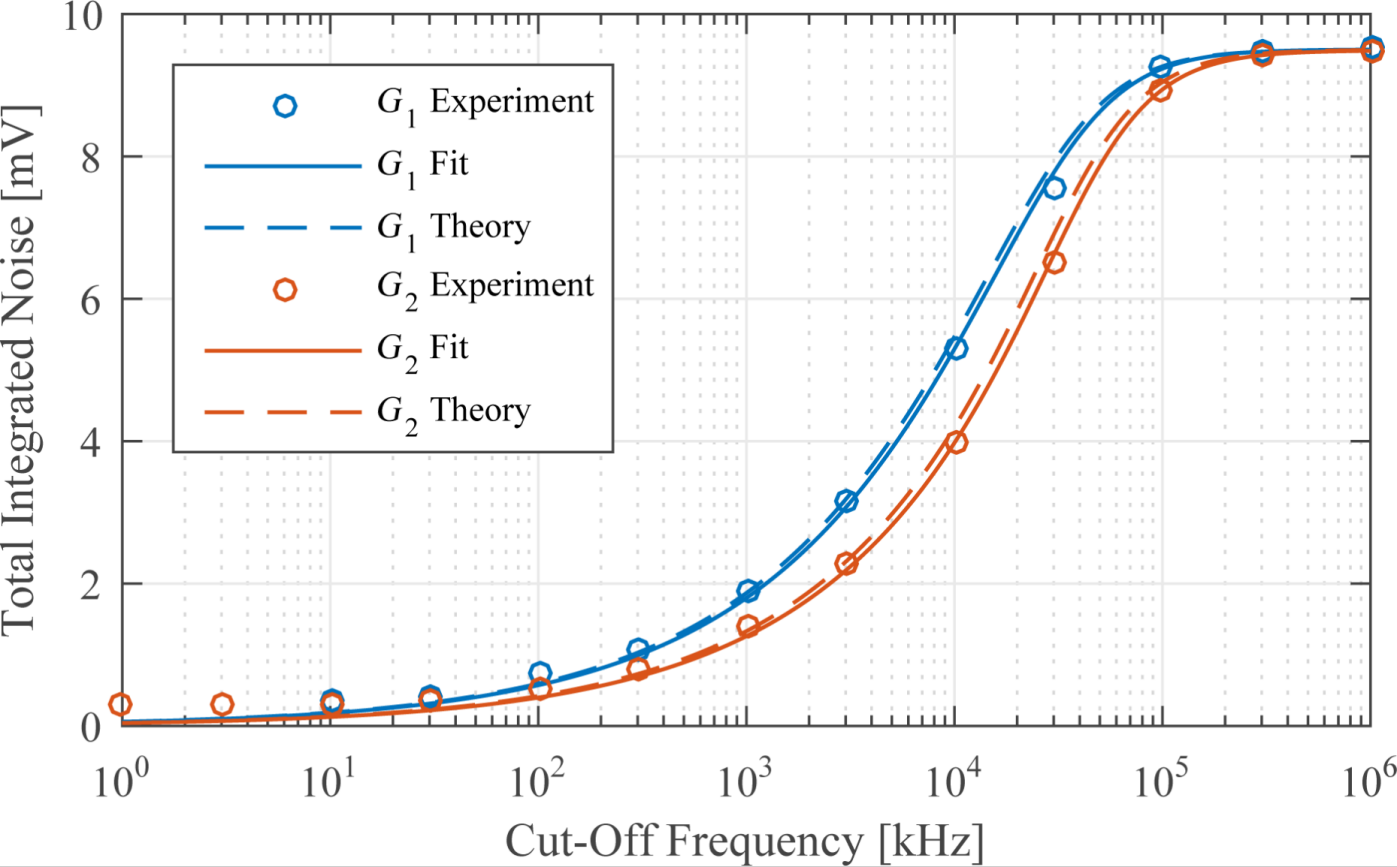
Experimental and theoretical total integrated noise of a low-pass filtered white noise process for a first-order system *G*_1_ and a second-order system *G*_2_. The experimental data was fitted to Equation 26 and Equation 29 using nonlinear least squares.

In Figure 23, the analytical Equations 26 and 29 are plotted against experimental low-pass filtered white noise processes. For this purpose, a function generator (Agilent 33500B Waveform Generator) was used to generate 40 kHz bandwidth limited white noise, which was subsequently low-pass filtered with a low-noise voltage preamplifier with variable cut-off frequency (Stanford Research SR560). The output of the filter was captured in the time-domain, sampled at *f_s_* = 2.56 MHz for *T* = 13.11 s, with the acquisition front end of a micro system analyzer (Polytec MSA-050-3D). The total integrated noise, is obtained by integrating the noise density from 0 to *f_s_*/2 using Welch’s method. It can be seen that for both systems, theory and experiment match very well. In theory the total integrated noise approaches zero as the filter bandwidth goes to zero. This discrepancy between theory and experiment can be attributed to digital noise, residual DC-offsets and 1/*f* noise in the signal.

## Acknowledgements

This research was performed at The University of Newcastle, Callaghan, NSW, Australia.
